# Transparent and luminescent glasses of gold thiolate coordination polymers†
†Electronic supplementary information (ESI) available: Chemicals, experimental details, and transmittance, PXRD, PDF, FT-IR, TGA, DSC, TMA, photoluminescence analysis and XANES results. See DOI: 10.1039/d0sc02258f


**DOI:** 10.1039/d0sc02258f

**Published:** 2020-06-09

**Authors:** Shefali Vaidya, Oleksandra Veselska, Antonii Zhadan, Maria Diaz-Lopez, Yves Joly, Pierre Bordet, Nathalie Guillou, Christophe Dujardin, Gilles Ledoux, François Toche, Rodica Chiriac, Alexandra Fateeva, Satoshi Horike, Aude Demessence

**Affiliations:** a Univ Lyon , Université Claude Bernard Lyon 1 , CNRS , Institut de Recherches sur la Catalyse et l'Environnement de Lyon (IRCELYON) , Villeurbanne , France . Email: aude.demessence@ircelyon.univ-lyon1.fr; b ISIS Facility , STFC Rutherford Appleton Laboratory , Didcot OX11 0QX , UK; c Diamond Light Source Ltd , Diamond House, Harwell Science and Innovation Campus , Didcot OX11 0DE , UK; d Univ Grenoble Alpes , CNRS , Institut Néel , Grenoble , France; e Université Paris-Saclay , UVSQ , CNRS , UMR 8180 , Institut Lavoisier de Versailles , 78000 , Versailles , France; f Univ Lyon , Université Claude Bernard Lyon 1 , CNRS , Institut Lumière Matière (ILM) , Villeurbanne , France; g Univ Lyon , Université Claude Bernard Lyon 1 , CNRS , Laboratoire des Multimatériaux et Interfaces (LMI) , Villeurbanne , France; h Institute for Integrated Cell-Material Sciences (WPI-iCeMS) , Institute for Advanced Study , Kyoto University , Yoshida-Honmachi, Sakyo-ku , Kyoto , Japan; i Institute of Experimental and Applied Physics , Czech Technical University in Prague , CZ-11000 Prague , Czech Republic

## Abstract

Obtaining transparent glasses made of functional coordination polymers (CPs) represents a tremendous opportunity for optical applications. In this context, the first transparent and red-emissive glasses of gold thiolate CPs have been obtained by simply applying mechanical pressure to amorphous powders of CPs. The three gold-based CP glasses are composed of either thiophenolate [Au(SPh)]_*n*_, phenylmethanethiolate [Au(SMePh)]_*n*_ or phenylethanethiolate [Au(SEtPh)]_*n*_. The presence of a longer alkyl chain between the thiolate and the phenyl ring led to the formation of glass with higher transparency. The glass transitions, measured by thermomechanical analysis (TMA), occurred at lower temperature for CPs with longer alkyl chains. In addition, all three gold thiolate glasses exhibit red emission at 93 K and one of them, [Au(SMePh)]_*n*_, remains luminescent even at room temperature. An in-depth structural study of the amorphous gold thiolates by XRD, PDF and EXAFS analysis showed that they are formed of disordered doubly interpenetrated helical chains. These d^10^ metal-based compounds represent the first examples of transparent and luminescent CP glasses.

## Abstract 2

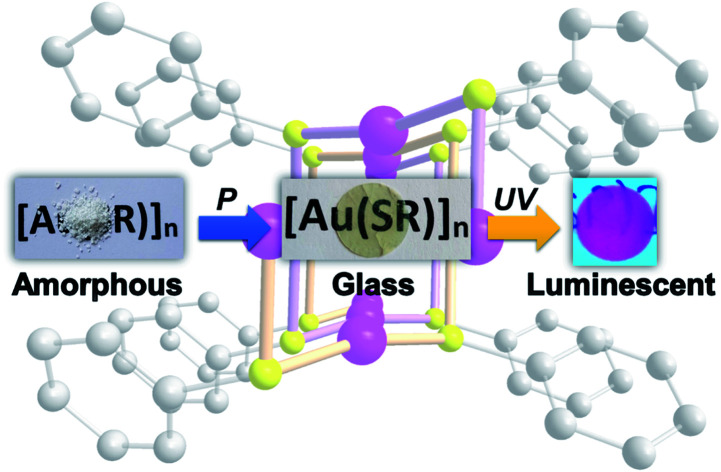
Low mechanical pressure on amorphous gold thiolate coordination polymers allows the formation of transparent and red emissive glasses.

## Introduction

Liquid, amorphous, and glass coordination polymers (CPs) and metal–organic frameworks (MOFs) have attracted great interest due to their mechanical properties, which are halfway between those of brittle inorganic glasses and ductile organic polymers.^1^ The association of the intrinsic physical properties of CPs, such as porosity, conductivity, magnetism or photoluminescence, with the possibility of shaping the materials as transparent glasses opens up new areas of applications in display and communication technologies.^2^ Still, a limited number of glasses of CPs have shown potential applications for molecule trapping and conservation^3^ and for ion transport in energy devices.^4^

Today, all the glass CPs reported in the literature are obtained *via* a two-step route that requires the synthesis of a crystalline CP, followed by ball milling and pressurization,^5^ heating or melt quenching^6^ of the crystalline phase.1*b* Most of the melt quenching approaches require the use of high temperature (between 400 and 600 °C) in the case of zeolitic imidazolate frameworks (ZIFs).1a,^7^ Moreover, the formation of large scale and transparent glasses of CPs is still challenging because of the difficulty in amorphizing polycrystalline phases and the harsh conditions that are required from an amorphous phase, such as 4 GPa at 70 °C for 2 hours.^8^

Some of us reported a simple one-step synthesis of an amorphous gold(i) thiophenolate CP, which undergoes a solid state amorphous-to-crystalline phase change upon heating.^9^ The first advantage of the d^10^ coinage metal chalcogenolates (MOCs) is their strong covalent soft–soft bonds between the d^10^ coinage metal (Cu, Ag, or Au) and the chalcogenolate (SR, SeR, or TeR), leading to CPs with good thermal and chemical stabilities,^10^ as opposed to the hard–hard interactions between oxygen atoms (carboxylates or phosphonates) and high oxidation state metals.^11^ This strong interaction and the μ_2_-bridging mode of the thiolate to Au(i) give access to wider bonding geometries, providing flexibility10*b* and favoring phase changes. The second benefit of the coinage MOCs is their ability to exhibit various photoluminescent properties due to the presence of d^10^ coinage metals and some metallophilic interactions.^11^ The peculiar optical properties displayed by anisotropic 1D and 2D MOCs have demonstrated their great potential for temperature sensing and optoelectronic applications.^12^ They are thus a good alternative to replace the lanthanide-based luminescent materials considered as critical elements and even banned by the European Union.^13^

**Fig. 1:**
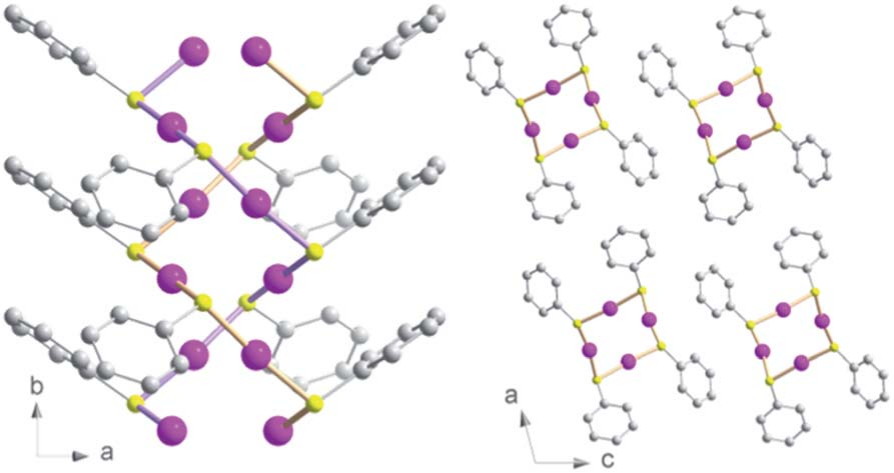
Views of the crystallographic structure of the [Au(SPh)]_*n*_ CP along the *c* axis (left) and *b* axis (right). Pink, yellow and grey spheres are for gold, sulfur and carbon atoms. Hydrogen atoms are omitted for clarity.

**Scheme 1:**
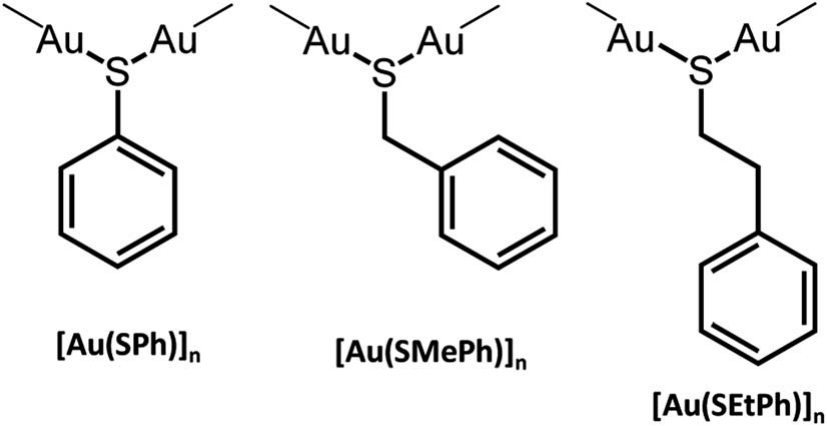
Presentation of the three [Au(SR)]_*n*_ compounds.

The formation of new CP glasses is based on our previous study on [Au(SPh)]_*n*_.^9^ In its crystalline form, obtained under hydrothermal conditions, [Au(SPh)]_*n*_ displays a 1D structure, consisting of two interpenetrated spiral chains of –Au–S–Au–S– that form a double helix (Fig. 1).^9^ The self-assembly is governed by aurophilic Au···Au and C–H···π interchain interactions. This crystalline phase is red emissive at room temperature originating from a Ligand-to-Metal–Metal Charge Transfer (LMMCT) and the radiative process implies the participation of the aurophilic interactions. On the other hand, under mild synthetic conditions, the [Au(SPh)]_*n*_ amorphous phase is obtained and a solid state amorphous-to-crystalline phase change occurs upon heating. Then, an in-depth study of the amorphous phase allowed us to highlight the possibility of transparent glass formation *via* a simple mechanical pressurization. This study was further extended to two other gold thiolates based on methylthiophenolate and ethylthiophenolate ligands (Scheme 1).

Here we present the first examples of the formation of transparent and luminescent glasses upon a soft mechanical pressurization of 1D amorphous gold thiolate CPs. Previous reports have only shown the possibility to form transparent and luminescent glasses with molecular lanthanide complexes.^14^ In addition, thermomechanical analysis (TMA) has been used for the first time for CP glasses to determine the glass transitions more precisely than in the case of Differential Scanning Calorimetry (DSC). In-depth structural characterization on the glass phases allowed us to describe a double-helix model.

## Results and discussion

### Fabrication of transparent glasses

**Fig. 2:**
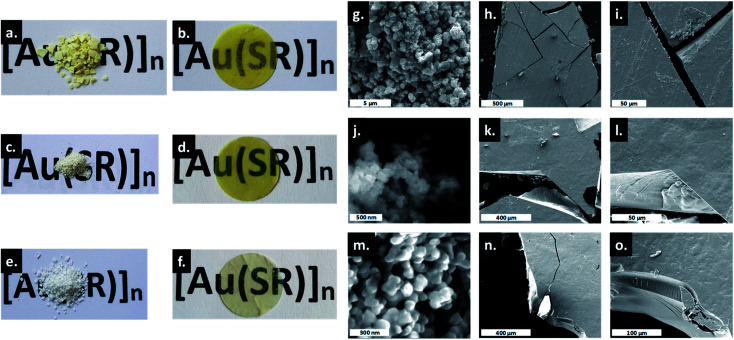
Photos of the powdery and glassy [Au(SR)]_*n*_: (a) **1a**, (b) **1g**, (c) **2a**, (d) **2g**, (e) **3a** and (f) **3g**. The diameter of the pellet is 13 mm. SEM images of **1a** (g), **1g** (h and i), **2a** (j), **2g** (k and l), **3a** (m) and **3g** (n and o).

The amorphous gold thiolate compounds were synthesized directly from a redox reaction between HAuCl_4_ and an excess of the corresponding thiol molecules, thiophenol (HSPh), phenylmethanethiol (HSMePh) and phenylethanethiol (HSEtPh) in methanol, at room temperate or 60 °C. These conditions are similar to the first step synthesis of gold thiolate clusters.^15^ Then, 50 mg of the obtained pale yellow and white amorphous powders, a-[Au(SR)]_*n*_, **1a**, **2a** and **3a** for a-[Au(SPh)]_*n*_, a-[Au(SMePh)]_*n*_ and a-[Au(SEtPh)]_*n*_, can be mechanically pressed at 10 tons under ambient atmospheric conditions to form transparent glasses, g-[Au(SR)]_*n*_, **1g**, **2g** and **3g** for g-[Au(SPh)]_*n*_, g-[Au(SMePh)]_*n*_ and g-[Au(SEtPh)]_*n*_, respectively (Fig. 2a–f). The thicknesses of these pellets are 140, 155 and 156 μm, respectively. While **1g** is a yellow translucent glass, **2g** and **3g** are yellow and colorless transparent glasses. Thus, the gain in the color and the transparency of the vitreous pellets appears to be related to the addition of the –CH_2_– motif between the thiolate and phenyl functionalities and the flexibility of the ligands.

The transmittance curves of the three g-[Au(SR)]_*n*_ pellets show that **1g** and **2g** start to let the light go through from 430 nm and reach a maximum transmittance of 19% at 850 nm, while **3g** starts to be transparent from 400 nm and reaches a maximum transmittance of 26% at 850 nm (Fig. S1†). This analysis proves the better transparency of **3g** compared to the two other samples. The relatively low percentage of transmittance, compared to that of silica glass, which reaches 100%, can be explained by the reflection at the interfaces and the scattering by defects at the microscale. In addition, when thicker pellets are prepared with 100 mg, the thicknesses are 295, 300 and 316 μm for **1g** to **3g** and the resulting transmittance is quasi null. A solution to increase the transparency would be to increase the applied pressure.

**Fig. 3:**
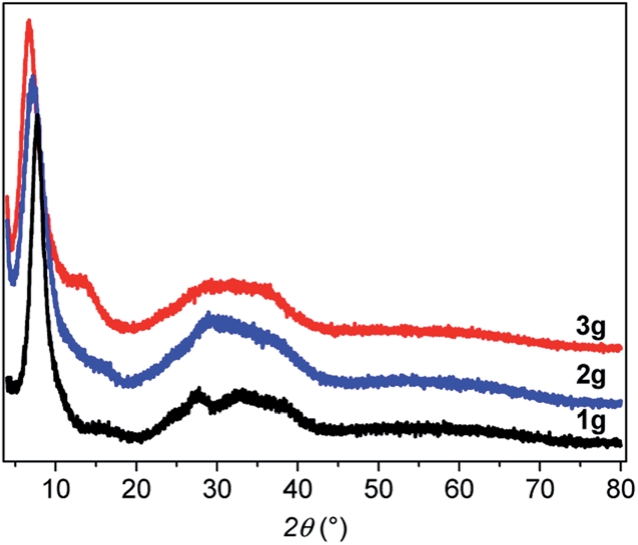
PXRD patterns of the three g-[Au(SR)]_*n*_.

From powder X-ray diffraction (PXRD), the three powder and pellet samples exhibit patterns with similar amorphous features (Fig. S2–S4†). After pressing the powder, no phase changes are observed in the PXRD patterns as has been seen for some Au(i) complexes upon mechanical grinding.^16^ The PXRD patterns (Fig. 3) show a sharp peak at low angle and a broad feature at 2*θ* between 20 and 40° characteristic of poorly crystallized (or non-crystalline) materials that will be called amorphous here. Similarly, the Pair Distribution Functions (PDFs) of the powder and pellet forms of [Au(SR)]_*n*_ do not show any difference, meaning that there is no change of the local or average structure when the powder is pressed (Fig. S5†). In-depth studies of the PDF data will be discussed in the next part.

Thus, the only evident change is the morphological aspect of the samples. The macroscopic texture of the powder and glass forms of [Au(SR)]_*n*_ solids was studied by SEM (Fig. 2g–o). As powders, the compounds form large aggregates of around 500 nm for **1a** (Fig. 2g), and well-defined spherical nanoparticles of around 80 and 50 nm for **2a** (Fig. 2j) and **3a** (Fig. 2m), respectively. Thus, the formation of smaller particles in the case of [Au(SEtPh)]_*n*_ may also be related to the better transparency of the glass. The SEM images of g-[Au(SR)]_*n*_ show a morphology characteristic of glass materials: flat surfaces with clear cracks and the absence of grain boundaries.

### Samples characterization

The presence of thiolates in the amorphous samples was confirmed by FT-IR spectroscopy exhibiting well defined bands of aromatic rings and alkane functionalities (Fig. S6†). From the thermogravimetric analysis (TGA) carried out in air, the weight loss of the amorphous phases corresponds well to the expected 1 : 1 ratio of ligand : gold (Fig. S7†). These experiments also show that **1a**, **2a** and **3a** start to decompose at 240, 184 and 225 °C, respectively (Table S1†). Thus **2a** is the less thermally stable compound as a powder. For **1g**, **2g** and **3g**, the decomposition begins at 225, 185 and 185 °C, meaning that the two more stable compounds, **1** and **3**, become less thermally stable in their glassy state, while **2** exhibits a constant thermal stability in the powdery and glassy forms.

**Fig. 4:**
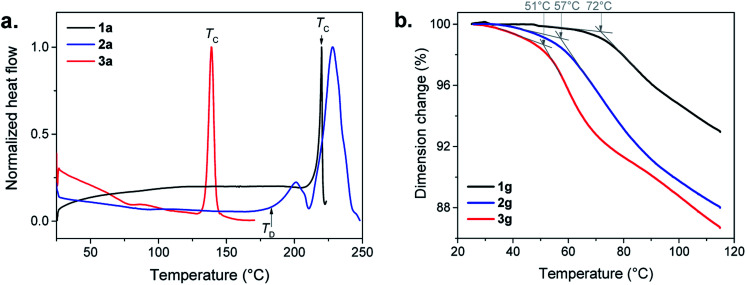
(a) DSC in air with a heating rate of 10 °C min^–1^, of the three amorphous a-[Au(SR)]_*n*_ compounds: **1a** (black), **2a** (blue) and **3a** (red), showing the crystallization temperature (*T*_C_) or the decomposition temperature (*T*_D_). (b) TMA curves of **1g** (black), **2g** (blue) and **3g** (red) carried out in air with a heating rate of 3 °C min^–1^ and an applied force of 0.01 N. The grey arrows and the associated temperatures correspond to the onset of the *T*_g_.

In the Differential Scanning Calorimetry (DSC) curves, before their decomposition, **1a** and **3a** exhibit an intense exothermic peak of crystallization (*T*_C_) at 220 and 140 °C, respectively (Fig. 4a, S8, S9 and Table S1†). The solid state amorphous-to-crystalline phase change of [Au(SPh)]_*n*_ from **1a** to **1c** has been reported.^9^ In the case of **3a**, the formation of a crystalline phase has also been observed (Fig. S10†), while, for **2a**, due to its lower thermal stability, no crystallization peak is observed before its decomposition (Fig. S11†).

The assignments of the glass transition temperature (*T*_g_) have first been made by DSC on the three **1a**, **2a** and **3a** samples (Fig. 4a). The *T*_g_ of **1a** and **2a**, when heated at 10 °C min^–1^, cannot be determined with accuracy because of the slight increase of the DSC signals due to an expansion of the samples during the glass transition. For **3a** the glass transition is more pronounced and the onset *T*_g_ is observed at 56 °C (Fig. S12†). Additional thermomechanical analysis (TMA) has been performed on the pellet samples (glass). Indeed, the TMA technique is better suited for *T*_g_ determination than DSC due to its high sensitivity to volume changes during thermal expansion (the material becomes less rigid in the transition from glassy to rubbery behavior). Thus, TMA measures material deformations under controlled conditions of force (here in compression mode at 0.01 or 0.1 N), temperature (25 to 120 °C) and heating rate (3 or 5 °C min^–1^). Fig. 4b shows clear *T*_g_ values for the three pellets of g-[Au(SR)]_*n*_ samples that correspond to the slope change of the dimension change curve for a heating rate of 3 °C min^–1^ and a force of 0.1 N. The onsets of the *T*_g_ decrease from 72 to 57 and 51 °C with the increase of the alkyl chain in the ligand (Fig. S13–S15†). When a faster heating rate is used at the same applied force (0.1 N), the onset of the *T*_g_ is shifted towards higher temperatures for **1g** (79 °C) and **2g** (68 °C), while the one for **3g** (48 °C) does not change as much (Fig. S16–S18†). Then a weaker force was applied (0.01 N) at 3 °C min^–1^ (Fig. S16–S18†). The dimension change is less important at this force for **1g** (1%) and **2g** (3%), but it is 10% for **3g** with a *T*_g_ onset that is well-defined at 53 °C. This clearly shows that amorphous [Au(SEtPh)]_*n*_ is more suitable to undergo the glass transition and it justifies also that the corresponding glass is the most transparent one.

The gradual improvement of transparency and the lowering of glass transition temperatures from **1a** to **3a** imply that the glass transitions are governed by the flexibility of the ligand associated with the length increase of the aliphatic chain. Indeed, a more flexible backbone (from **1a** to **3a**) induces more movements, *T*_g_ at lower temperature and better transparency. These three compounds still have short chains, and each of the ligands can be considered as discrete entities with their own environment and behavior.^17^ In contrast, crystalline layered copper,^18^ silver^17^,^19^ and gold^20^*n*-alkanethiolates are known to melt due to the presence of long chains, while the strong M–S bonds remain intact.

From the PXRD, the *d* spacing of the first peak in Fig. 3 corresponds to the distance between the helical chains. The increased distances of 1.14, 1.23 and 1.31 nm for **1a**, **2a** and **3a**, respectively, can be correlated with the increasing length of the ligands with stacking of the organic moieties without interpenetration of the aromatic rings (Fig. S19†).

### Photoluminescent properties

**Fig. 5:**
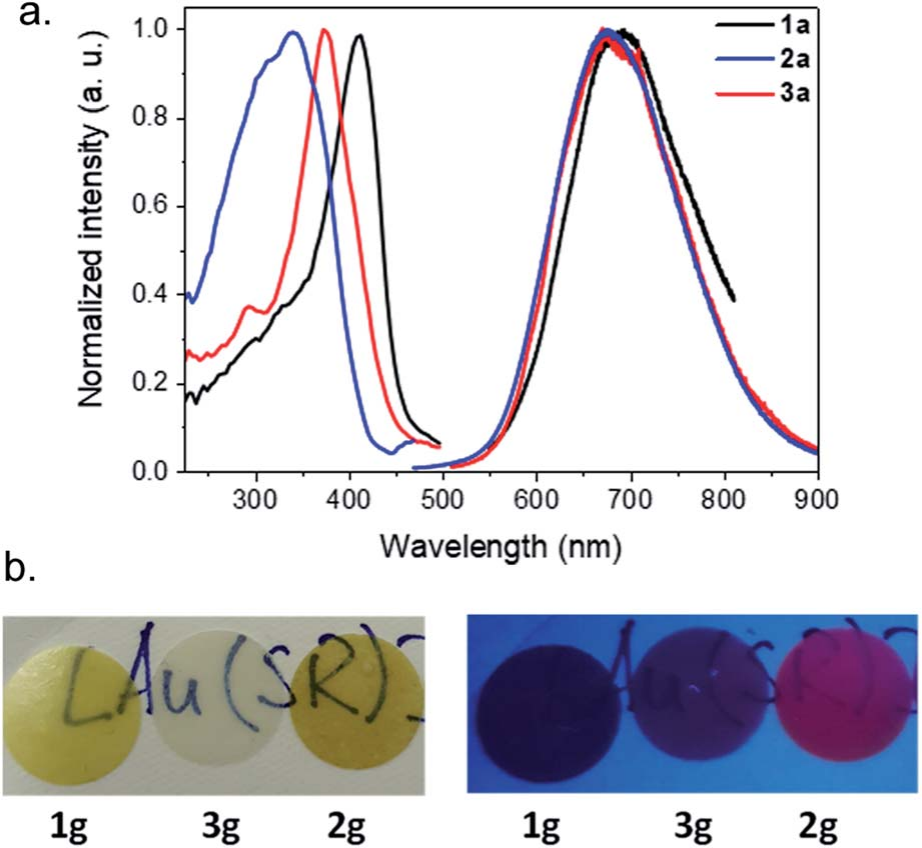
(a) Emission–excitation spectra of **1a** (black), **2a** (red) and **3a** (blue) obtained in the solid state at 93 K with, respectively, (*λ*_ex_, *λ*_em_) in nm as follows: (412, 690), (372, 675) and (340, 675). (b) Photos of g-[Au(SR)]_*n*_ pellets at room temperature under normal light (left) and under a UV lamp (right).

Bearing in mind that the crystalline [Au(SPh)]_*n*_ CP (**1c**) is a luminescent material, the photophysical properties of the amorphous phases have been studied as a function of the temperature. As mentioned before, **1a** is non-emissive at room temperature. However, it becomes emissive below 250 K, and the intensity of emission increases with lowering the temperature (Fig. 5a, S20 and S21†). This intensification is due to the enhanced rigidity of the network and the faster intersystem crossing process at low temperature, reducing the energy loss by non-radiative decay. The position of the maximum of emission is slightly red shifted from 675 nm for **1c** (*λ*_ex_ = 320 nm) to 690 nm for **1a** (*λ*_ex_ = 412 nm) (Fig. S22†). In addition, the emission peak at 93 K of **1a** is wider than the one of **1c** (the width at half maximum is 63 nm (1359 cm^–1^) *vs.* 165 nm (3371 cm^–1^), respectively). The Stokes shift is different too – 9780 and 16 435 cm^–1^ for **1a** and **1c**, respectively (Table S2†). The lifetime decay at 93 K of **1c** has one major contribution of 8.4 μs (98%), while **1a** exhibits a long decay of 9.9 μs (71%) and a shorter one of 0.1 μs (29%) (Fig. S23 and Table S3†). So, the large Stokes shift and microsecond lifetime decays support the presence of a phosphorescence process in **1a** that can be close to that in **1c**. However, the less intense emission of **1a** associated with two lifetime decays can be explained by (i) the trapping of energy on the defects that are omnipresent in amorphous materials and/or (ii) a reduced number of aurophilic interactions, that can be related to the aggregation induced emission observed for some Au(i) thiolate clusters and complexes.^21^

Similarly to **1a**, **3a** is not luminescent at room temperature, while **2a** is weakly red emissive (Fig. S24†). The previous analyses carried out show that the three amorphous samples are quite similar; a tentative explanation for the presence of the emission of **2a** at RT could be related to some different electronic transitions induced by the ligand. At 93 K, **2a** and **3a** possess a close red emission centered at 675 nm and an important Stokes shift > 12 000 cm^–1^ (Fig. 5a and S25, and Table S2†). The two-component lifetime decays of **2a** are 0.8 μs (88%) and 73 μs (12%) and those of **3a** are 7.7 μs (82%) and 0.2 μs (18%), indicating phosphorescence mechanisms (Table S3 and Fig. S26†). The shift of the positions of the excitation peak centered at 412, 372 and 340 nm for the three samples from **1a** to **3a** can be explained by the different distributions of the electronic density of the HOMO level on the different organic ligands (Fig. 5a).

After the pelletizing process, the vitreous samples retain their photophysical properties (Fig. 5b, S23–S25, and S27–S29, and Tables S2 and S3†), which confirms that there is no structural modification upon pressurization. In addition, due to the stability of gold thiolate coordination polymers,10*b***2g** glass remains luminescent at RT after 1.5 years storage under ambient storage conditions (Fig. S30†). Pellet **2g** is the first example of a transparent and RT luminescent glass made of pure coordination polymers (Fig. 5b).

In order to gain in-depth structural information to try to explain the different *T*_g_ and luminescent properties, Pair Distribution Function (PDF) and Extended X-ray Absorption Fine Structure (EXAFS) analyses were carried out.

### PDF analyses

The structural characterization of these samples in reciprocal space is greatly challenged due to the poor crystallinity which results in broad features in the PXRD patterns. To overcome this difficulty, we analyzed the PDF obtained by total scattering measurements. The PDF represents the probability of finding a pair of atoms at a given distance *r* in the sample, and yields structural information in direct space on the local atomic arrangement. The PDF was shown to provide important information on the structure of complex non-crystalline compounds such as amorphous inorganic zeolites^22^ and MOFs,^23^ which is difficult to obtain by other techniques. One of the main targets here is to distinguish if the amorphous phases are built of continuous molecular networks or of isolated complexes. The PDF of the known crystalline [Au(SPh)]_*n*_ (**1c**) is first reported and compared to that of its amorphous counterpart (**1a**). Finally, the PDFs of **2a** and **3a** are compared to that of **1a**.

**Fig. 6:**
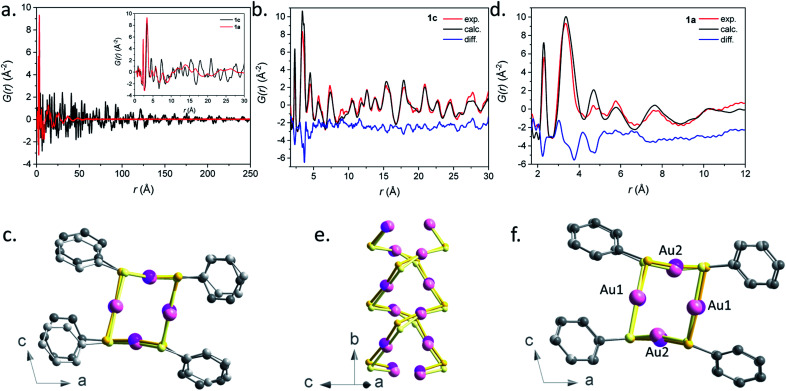
(a) Pair distribution function *G*(*r*) for samples **1a** (red) and **1c** (black). The short *r* part of the PDFs up to 30 Å is shown in the inset. (b) Result of the PDF refinement using the MolPDF software for the **1c** sample: experiment (red), calculation (black) and difference (blue). (c) Common projection of the original structure from ref. 9 (light color atoms) and the one of sample **1c** refined with MolPDF along the *b* axis. (d) Result of the PDF refinement using the MolPDF software for the **1a** sample with the double-helix model: experiment (red), calculation (black) and difference (blue). Common projections of (e) **1a** (light color atoms) and **1c** samples with the double-helix models both refined with MolPDF and (f) the structures of samples **1c** and **1a** (light color atoms) both refined with MolPDF along the *b* axis. Pink, yellow and grey spheres are for gold, sulfur and carbon atoms.

The PDF *G*(*r*) and reduced structure functions *F*(*Q*)^24^ of samples **1c** and **1a** are shown in Fig. 6a and S31.†

A qualitative inspection of Fig. 6a first indicates that sample **1c** is crystalline with well-defined Bragg peaks. PDF oscillations extend over 250 Å, indicating that the size of the structure coherence domains exceeds this value. The observed decay of the PDF with *r* is essentially due to the effect of experimental spatial resolution. For **1a** the Bragg peaks in *F*(*Q*) are replaced by broad oscillations, characteristic of amorphous or disordered materials. Due to the large contrast in scattering power between the organic molecule and metal atoms, the diffraction and PDF signals are strongly dominated by the scattering of gold and to a lesser extent sulfur atoms. In fact, as can be seen in Fig. S32,† most features of the PDF correspond to the contribution of Au–Au atomic pairs, with only the first peak at 2.3 Å being solely due to Au–S pairs. In the PDFs of **1a** and **1c**, this first peak is almost identical for the two samples, indicating that Au–S bond lengths are not modified between the crystalline and amorphous compounds (Fig. S33 and Table S4†). At longer distances between 3 and 9 Å, the relatively well-defined peaks are essentially due to Au–Au distances of gold atoms bridged by a thiolate molecule and the aurophilic interactions between the chains. Despite some similarities, one can observe marked differences between the PDFs of **1a** and **1c** in this distance range, implying some structural reorganization on the local scale (Fig. S33†). At longer distances, these well-defined oscillations in **1c** are replaced by much broader and roughly periodic oscillations in **1a** that progressively decrease in amplitude up about 50 Å (Fig. 6a). Their approximate period of ∼11.5 Å is close to the distance between the double helix chains in the crystalline compound, suggesting that these chains conserve some positional correlation in the amorphous phase up to several tens of angstroms. On the other hand, the structural coherence along them is much shorter (up to 9 Å) (Fig. S33 and Table S4†). In order to verify that the crystal structure of **1c**, determined by standard crystallographic techniques, is also valid at the local scale, we have performed a refinement of its PDF using MolPDF software^25^ and of complementary Au L_3_ edge EXAFS data providing fine details of the partial pair distribution function of atoms surrounding gold.

The MolPDF software allows applying restraints on bond distances and angles, which is necessary to reduce the number of refined parameters while maintaining the configuration of the molecules within the structure, especially in the presence of heavy atoms such as gold. The crystallographic structure of **1c** in space group *C*2/*c* was used as a starting model for the refinement, disregarding the hydrogen atoms.^9^ The first neighbor C–C distances and the C–C–C and C–C–S angles were constrained to their starting values. Atoms of the same elements had equal isotropic thermal parameters, which were varied as well as the atomic positions, cell parameters, scale factor and a sharpening factor. The last constraint was applied to the restrained C–S and C–C bonds and allows us to consider the difference between the Debye–Waller factor of intramolecular and other distances. The refinement was carried out between 1.7 and 30.0 Å. The results are reported in Table S4† and the refinement plot in Fig. 6b, which shows a quite satisfactory agreement for this type of soft material with distances between the refined positions and the original ones from ref. 9 not exceeding 0.2 Å for Au and S atoms. The main differences consist of relatively small rotations of the molecules as seen in Fig. 6c, which are difficult to confirm given the weak contribution of the molecules to the refined patterns both for the present PDF refinement and the original Rietveld refinement of ref. 9. However, the present PDF analysis confirms the validity of the crystallographic structure at the local scale.

The refined structure of **1c** was used as a starting point for the refinement of the structural model of the amorphous phase **1a** keeping the *C*2/*c* monoclinic symmetry and applying the same type of structure constraints as for sample **1c**. The refinement was carried out between 1.7 and 12.0 Å, which only corresponds to distances within a double helix chain. The results of the refinement are given in Fig. 6d and Table S4.† Although the agreement is poorer than in the case of the crystalline sample, the fit reproduces reasonably well all Au–S and Au–Au distance peaks up to ∼9 Å, indicating the preservation of the double helix chain coherence up to two unit cells along the *b* axis on average. This length corresponds to six gold atoms along one helix (Fig. 6e). One can propose that the Au–S covalent bonds would tend to preserve the structural coherence along the chains while disorder would be introduced through displacements/rotations between the molecules weakly linked by parallel π interactions. The largest local structure modification with respect to the crystalline phase is a displacement of ∼0.3 Å of both Au atoms in the (*b*,*c*) plane leading to a decrease of the double helix distortion. The difference between the longer Au2–Au2 distances (3.81(3) Å) and the shorter Au1–Au1 ones (3.27(4) Å) for sample **1a** is much smaller than for sample **1c** (4.16(1) Å and 3.36(1) Å, respectively) (Fig. 6f). The slight decrease of the Au–Au distances in **1a**, that induces stronger aurophilic interactions, does not correlate with the less intense photoemission of **1a** compared to **1c**.^26^ So, as already stated in the photoluminescent analysis part, the less effective photoemission of **1a** may be due to (i) the trapping of energy on the defects and/or (ii) a reduced number of aurophilic interactions.

The PDF data of the amorphous **1a** phase were also refined with a model consisting of tetramer rings, since gold thiolates are known to form cyclic structures with bulky ligands,^27^ and several tetrameric oligomers, [Au(SR)]_4_, have been reported as intermediates in the formation or fragmentation of gold thiolate clusters.^28^ The distorted 8-membered ring model was built based on the refined double-helix model of **1c**, by the displacement of half of the thiophenolate molecules by 0.5 f. u. along the *b*-axis (Fig. S34†). In the tetrameric ring and double-helix models, the atomic positions of gold atoms are preserved. The main difference is the more acute S–Au–S angles of the tetrameric ring – 104 and 140° (*vs.* 159 and 166°, see Table S5†). Despite the fact that the nearest Au–S and Au–Au distances corresponding to the contacts inside the ring are well described by this model, it fails to reproduce longer correlations at 4.5 and 7.7 Å corresponding to Au–Au contacts between neighboring tetramers (Fig. S35 and S36†) and the refinement yields poorer agreement factors.

Therefore, our refinements indicate that for the amorphous sample **1a**, the connectivity of Au–S corresponds to double helices with a coherence length of ∼9 Å and not to tetrameric species. This hypothesis is supported by the insolubility of **1a**, since small oligomeric molecules are likely to be soluble.^29^ However, the presence of tetrameric motif defects intercalated in a double helix could occur, which would reduce the coherence length.

All three **1a**, **2a** and **3a** amorphous compounds show PDFs with similar features (Fig. S37†). The peaks corresponding to the first Au–S and Au–Au are positioned at 2.3 at 3.5 Å for all three materials. Thus, one can propose that these three amorphous phases are formed with a disordered double-helix Au(i)–S core. At long *r*, only broad features are observed, which are damped out to disappear at around 50 Å (Fig. S37b†). Similarly to **1a**, this can be interpreted for **2a** and **3a** as due to the positional correlations between the double helices. Expectedly, the shift of these broad features to longer distances is related to the longer thiolate ligands in **2a** and **3a**.

### Au-L_3_ XAS study of crystalline (**1c**) and amorphous (**1a**) [Au(SPh)]_*n*_

Next, the Au-L_3_ EXAFS providing fine details of the partial pair distribution functions involving the absorber, *i.e.* Au, is introduced. As well as for PDF analysis, the interpretation of EXAFS does not make any assumption on the symmetry and periodicity of the structure, and the quantitative analysis of EXAFS provides accurate interatomic distances, coordination number and mean-square displacements for the nearest shells.

**Fig. 7:**
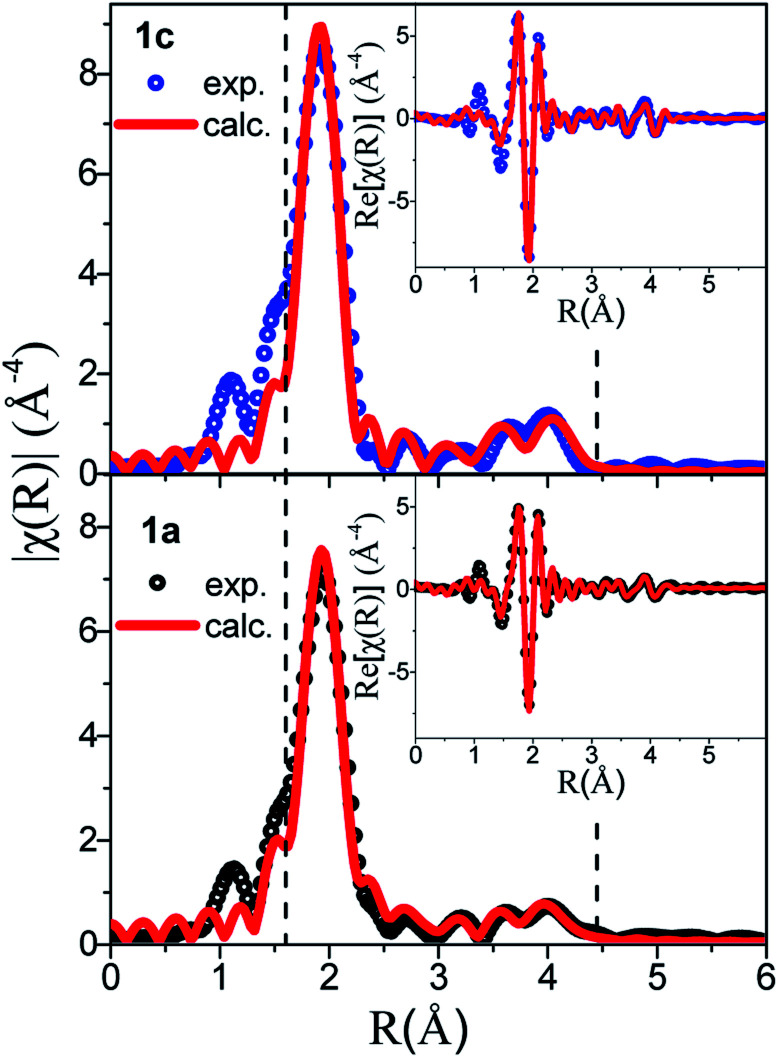
EXAFS fitting of samples **1c** (top) and **1a** (bottom) using the double-helix model.

The comparison between the *χ*(*k*) and FT *χ*(*R*) signals of **1a** and **1c** is given in Fig. 7, where the peaks corresponding to the three Au–S nearest paths have been highlighted. The loss of intensity in these peaks observed on the amorphous sample is correlated with the increased atomic disorder in **1a**. This observation is supported by the increased mean-square displacement (*σ*^2^) refined values reported in Table S6.†

Table S6† introduces the refined distances (*R*) and mean square deviations in distance (*σ*^2^) for the five innermost shells involving the coordinating species Au and S as well as the coordination numbers (*N*) of the first nearest S neighbor. Note that the contribution of the Au–C and Au–H partial pair distribution functions to the EXAFS signal is minor (the Au–C estimated contribution is <10% that of Au–S) and these paths were not included in the refinement.

The EXAFS data of **1c** were refined based on its known crystallographic structure, giving a good fit to the data (Fig. 7), and interatomic distances in good agreement with those from the PDF (Tables S2 and S6†). The EXAFS data of **1a** were also refined with the double-helix model, which also gave an excellent fit to the data and refined distances in good agreement with the PDF, albeit with a coordination number for the first S neighbor of only 1.5(1), lower than the expected value of 2. The lower number of S neighbors could be an indication of some breaking of the chains in the amorphous **1a**. A coherence length of six Au atoms along one helix chain as found by PDF analysis gives eight Au atoms with coordination number (CN) 2 and four with CN 1 providing an average coordination number of 1.7 close to the value refined by EXAFS. Unfortunately, the second model, the tetramer ring model, where only the distribution of the first nearest neighbors is known, could not be used to fit the EXAFS. Unlike in PDF, where peak positions are directly translated into interatomic distances, in EXAFS, the peak position also depends on the phase amplitude. Thus, when fitting EXAFS data a cluster of ∼9 Å around the absorber atom is considered in order to compute reasonably well the muffin tin potentials. A complex structural modelling approach based on the Reverse Monte Carlo Modelling of combined datasets is currently under investigation, which could allow for a full structural description in this disordered system.

The near-edge region of the spectra (XANES), giving a projection of the unoccupied density of states, was also evaluated. In 5d transition metals, 2p_3/2_ → 5d_5/2_, 5d_3/2_ dipole allowed transitions are responsible for the near-edge structure at the L_3_ absorption edge. Since this study deals with Au(i) compounds with electronic configuration [5d^10^6s^0^], a low intensity white line, similar to the one recorded for a metallic Au foil standard (Fig. S38†), has been observed, as expected.

**Fig. 8:**
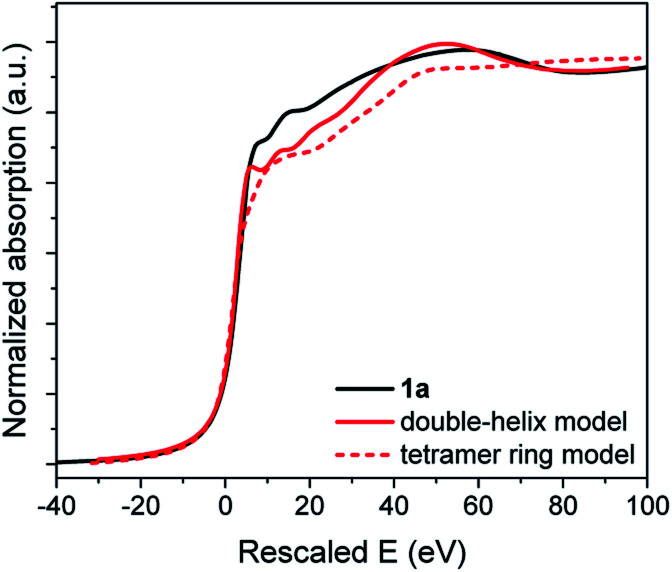
XANES data of **1a** (black) and simulations using the double-helix (solid red) and tetramer ring models (dotted red). The rescaled energy values are plotted in the X-axis, corresponding to the outputs of the FDMNES simulations.

A slight increase in intensity of the white line can be observed from the comparison between **1c** and **1a** XANES in Fig. S39.† With both compounds having the same oxidation state of Au and thiolate ligands, this difference is attributed to the different environments around the Au(i) and S–Au–S bonding geometries introduced earlier. We have applied first-principles calculations based on density functional theory optimized (FDMNES) for XAS analysis to compute the XANES region of the refined **1c** and **1a** models to compare with the experimental data in Fig. 8 and S39.† 30 In the XANES region, the photoelectron interacts with a large number of atoms: a cluster of 7.5 Å radius, taking into account interaction with 116 atoms, was considered in our calculations. Thus, we have used the agreement between the simulated and experimental data to measure the veracity of the proposed models for **1a**. Note that the double-helix model for crystalline Au-thiolate successfully reproduces the experimental XANES, validating this simulation approach (Fig. 8).

As for PDF analysis, the double-helix model provides a better description of the overall shape of the XANES spectra than a block model. In the former, the first peak at 7.92 eV after the edge and the valley at 80 eV are accurately reproduced, while these features are absent for the calculated tetramer ring model spectra.

## Conclusion

Three new glasses of gold(i) thiolate CPs, [Au(SPh)]_*n*_, [Au(SMePh)]_*n*_ and [Au(SEtPh)]_*n*_, have been presented. The direct synthesis of the amorphous phases and the easy formation of pellets by a simple low pressure mechanical action at room temperature allow formation of transparent and large glasses. Better transparency is obtained for [Au(SEtPh)]_*n*_ compared to the yellowish glasses of [Au(SPh)]_*n*_ and [Au(SMePh)]_*n*_. The TMA experiments, used for the first time in the CP domain, showed indeed that the glass transition happens at lower temperature when the alkyl chain length increases. Thus, the incorporation of flexibility in the thiolate ligand allows better optical quality of the glasses. In addition, these gold thiolate glasses are the first example of red emissive CP-based glasses. These luminescent properties are observed thanks to the presence of the d^10^ gold metal and the presence of aurophilic interactions that induce ligand-to-metal–metal charge transfer. Through PDF analysis of the amorphous [Au(SPh)]_*n*_ phase, a model of distorted doubly interpenetrated gold-thiolate chains is proposed. This study, supported with EXAFS, confirms the presence of a double-helix and invalidates the presence of tetramer ring motifs in these amorphous phases. The easy formation of these gold thiolate CP glasses, associated with their high optical quality and red emission, opens the path to numerous applications in the field of lightning and optoelectronic devices.

## Experimental section

### Synthesis of a-[Au(SPh)]_*n*_ (**1a**)

The same procedure as described previously was used.^9^

### Synthesis of a-[Au(SMePh)]_*n*_ (**2a**)

To a solution of HAuCl_4_·3H_2_O (100 mg, 1 eq., 0.253 mmol Au basis) in methanol (10 mL), phenylmethanethiol (178 μL, 6 eq., 1.518 mmol) was added, and the mixture was stirred for 18 hours at 60 °C. The white product was recovered by centrifugation (4000 rpm, 10 min) and washed with methanol and collected *via* centrifugation again. The last step was repeated three times in order to completely remove the salts and the disulfides formed. The amount of a-[Au(SMePh)]_*n*_ compound was 70 mg (yield of 75% based on gold).

### Synthesis of a-[Au(SEtPh)]_*n*_ (**3a**)

To a solution of HAuCl_4_·3H_2_O (200 mg, 1 eq., 0.506 mmol Au basis) in methanol (10 mL), phenylethanethiol (408 μL, 6 eq., 3.047 mmol) was added, and the mixture was stirred for 1 hour at room temperature. The white product was recovered by centrifugation (4000 rpm, 10 min) and washed with ethanol and collected *via* centrifugation again. The last step was repeated three times in order to completely remove the salts and the disulfides formed. The amount of a-[Au(SEtPh)]_*n*_ compound was 120 mg (yield of 61% based on gold).

### Formation of g-[Au(SR)]_*n*_ glasses

50 mg of the powdery a-[Au(SR)]_*n*_ solids were deposited between two 13 mm highly polished stainless steel disks and inserted in a Specac™ Atlas™ Evacuable Pellet Die, usually used for KBr pellet production for FTIR analysis samples. The pressure applied with the pressing system was 10 tons for 60 s at room temperature and under ambient atmosphere. Pellets with 100 mg of powder were also prepared under the same conditions. All the characterizations of the glasses, except for transmittance, were carried out on the ones fabricated with 50 mg of amorphous powder.

## Conflicts of interest

The authors declare no competing financial interest.

## Supplementary Material

Supplementary information
