# Supplementary material for: Transparent and luminescent glasses of gold thiolate coordination polymers
Source: Chem Sci. 2020 Jun 9;11(26):6815–23. doi: 10.1039/d0sc02258f (PMC7505088; doi:10.1039/d0sc02258f)
Supplement: Supplementary file 1 [file SC-011-D0SC02258F-s001.pdf]

Electronic Supplementary Material (ESI) for Chemical Science.
This journal is © The Royal Society of Chemistry 2020

Electronic Supporting Information for:

Transparent and luminescent glasses of gold thiolate coordination polymers

Shefali Vaidya,[a] Oleksandra Veselska,[a†] Antonii Zhadan,[a] Maria Diaz-Lopez,[b] Yves Joly,[c] Pierre Bordet,[c] Nathalie Guillou,[d] Christophe Dujardin,[e] Gilles Ledoux,[e] François Toche,[f] Rodica Chiriac,[f] Alexandra Fateeva,[f] Satoshi Horike,[g] and Aude Demessence*,[a]

[a] Univ Lyon, Université Claude Bernard Lyon 1, CNRS, Institut de recherches sur la catalyse et l'environnement de Lyon (IRCELYON), Villeurbanne, France.

[b] ISIS Facility, STFC Rutherford Appleton Laboratory Didcot OX11 0QX, UK and Diamond Light Source Ltd. Diamond House, Harwell Science and Innovation Campus Didcot OX11 0DE, UK.

[c] Université Grenoble Alpes, CNRS, Institut Néel, Grenoble, France.

[d] Université Paris-Saclay, UVSQ, CNRS, UMR 8180, Institut Lavoisier de Versailles, 78000, Versailles, France.

[e] Univ Lyon, Université Claude Bernard Lyon 1, CNRS, Institut Lumière Matière (ILM), Villeurbanne, France.

[f] Univ Lyon, Université Claude Bernard Lyon 1, CNRS, Laboratoire des Multimatériaux et Interfaces (LMI), Villeurbanne, France.

[g] Institute for Integrated Cell-Material Sciences (WPI-iCeMS), Institute for Advanced Study, Kyoto University, Yoshida-Honmachi, Sakyo-ku, Kyoto, Japan.

[†] Actual address: Institute of Experimental and Applied Physics, Czech Technical University in Prague, CZ-11000 Prague, Czech Republic.

*aude.demessence@ircelyon.univ-lyon1.fr

Chem. Sci.

Chemicals.

Thiophenol (HSPh, > 99 %) and phenylethanethiol (HSEtPh, 98 %) were purchased from Sigma Aldrich. Phenylmethanethiol (HSMePh, 96 %) was purchased from TCI. Tetrachloroauric acid trihydrate ($HAuCl_4 \cdot 3H_2O$, ≥ 49 % Au basis) was purchased from Alfa Aesar. Methanol and ethanol were purchased from VWR Chemicals. All reagents and solvents were used without further purification.

Experimental.

Routine PXRD

Routine powder X-ray diffraction (PXRD) was carried out by a Bruker D8 Advance A25 diffractometer using Cu Kα radiation equipped with a 1-dimensional position-sensitive detector (Bruker LynxEye). X-Ray scattering was recorded between 4° and 90° (*2θ*) with 0.02° steps and 0.5 s per step (28 min for the scan). Divergence slit was fixed to 0.2° and the detector aperture to 192 channels (2.95°).

FT-IR

The infrared spectra were obtained by a Bruker Vector 22 FT-IR spectrometer with KBr pellets at room temperature and registered from 4000 cm^{-1} to 400 cm^{-1}.

TGA

Thermo-gravimetric analyses (TGA) were realized on a TGA/DSC 1 STARe System from Mettler Toledo. Around 5 mg of sample was heated with a rate of 40 $°C.min^{-1}$, in a 70 μL alumina crucible, under the air atmosphere (20 $mL.min^{-1}$).

DSC

Differential scanning calorimetry (DSC) measurements were carried out using the DSC 1 from Mettler Toledo. The following experimental conditions have been considered: a sample mass of about 5 mg, an aluminium crucible of 40 μL with a pinhole, heating rate of 10$°C.min^{-1}$, a temperature range of 25–250°C and air atmosphere (30 $mL.min^{-1}$). The DSC device was calibrated over the range 25–400°C, and the melting points and enthalpies of standards (i.e. indium and zinc) were used for the calibration in terms of heat flow and temperature. To exploit the obtained thermograms, the Mettler Toledo STAR 10.1 software was used.

TMA

A TMA/SDTA 840 from Mettler-Toledo has been used for the TMA measurements. The samples have been heated between 30 and 120°C at a heating rate of 3 or 5°C/min under an applied force of 0.01 or 0.1 N. The sample has been inserted between two quartz disks and the obtained « sandwich » was directly put inside the sample holder. The measuring probe used to apply the force was of 3 mm ball-point type in quartz glass, because of its low coefficient of thermal expansion and its high thermal stability. TMA was used in compression mode under air, which allows to use a homogenous force on all the surface of the sample by means of the quartz disk between the probe and the sample.

SEM

SEM images were obtained by FEI Quanta 250 FEG scanning electron microscope. Samples were mounted on stainless pads and sputtered with Au/Pd alloy to prevent charging during observation.

Transmittance

Transmission measurement were performed on a Perkin Elmer UV/vis/NIR lambda 900 spectrometer with a limiting diaphragm of 1 mm and a resolution of 1 nm.

Photoluminescence measurements

The photoluminescence measurements were performed on a homemade apparatus. The sample was deposited on a silicon substrate to form a small mound of 4 mm in diameter and of ~1 mm in thickness. It was illuminated by an EQ99X laser driven light source filtered by a Jobin Yvon Gemini 180 monochromator. The exit slit from the monochromator was then reimaged on the sample by two 100m focal length, 2 inch diameter MgF_2 lenses. The whole apparatus has been calibrated by means of a Newport 918D low power calibrated photodiode sensor over the range 190-1000 nm. The resolution of the system is 4 nm. The emitted light from the sample is collected by an optical fiber connected to a Jobin-Yvon TRIAX320 monochromator equipped with a cooled CCD detector. At the entrance of the monochromator, various long pass filters can be chosen to eliminate the excitation light. The wavelength dependence of the detection system was previously calibrated using a NIST calibrated QTH 45W lamp. The resolution of the detection system is 2 nm.

Temperature control over the sample was regulated by a THMS-600 heating stage with T95-PE temperature controller made by Linkam Scientific Instruments.

Luminescence lifetime measurements

During the luminescence lifetime measurements compounds were excited by a diode pumped 50 Hz tunable OPO laser made by EKSPLA. The luminescence emitted by the sample was collected by an optical fiber and afterwards filtered by a long pass filter (by Thorlabs, FEL500) and fed to a R2949 photomultiplier tube from Hamamatsu. Photon arrival times were categorized by the MCS6A multichannel scaler from Fast ComTec.

The data could not be fitted by a sum of simple exponential decays. For this reason stretched exponential decay was used (Eq. 1):[1]

$$I = a_1 e^{-(x/t_1)^{\beta_1}} + a_2 e^{-(x/t_2)^{\beta_2}} \quad \text{(Eq. 1)}$$

Here, a_i and t_i are an amplitude and lifetime of a given component i and β_i is enclosed between 0 and 1.

The β factor is introduced into the function in order to account for possibility of energy transfer towards a distribution of non-radiative centers by dipole-dipole/quadrupole-quadrupole/etc. interactions. Its value is given by the type of interactions and the dimensionality of the system.

The average lifetime of the stretched exponential decay $< \tau_i >$ is calculated using the Equation 2. The procedure used for the fitting is described in [2].

$$< \tau_i > = \tau_i \cdot \frac{1}{\beta_i} \cdot \Gamma\left(\frac{1}{\beta_i}\right) \quad \text{(Eq. 2)}$$

For a multiexponential decay, by variation of the lifetime one achieves compensation of the amplitude and vice versa. It is possible to obtain similar decay intensity with different values of a_i and t_i. In other words, a_i and t_i are correlated. The situation turns even more complicated, once β_i is different from 1. The unfortunate result is that the ability to determine the precise values is greatly hindered by parameter correlation.[3] For this reason, some of the fit parameters should be considered carefully.

Total scattering measurements.

The powdered samples of **1a**, **1c**, **2a** and **3a** were loaded into 0.7 mm diameter borosilicate glass capillaries. The glass samples (**1g** and **3g**) were mounted on top of the goniometer in vertical position.

Room temperature X-ray data for samples **1a** and **1c** were collected at the ID22 beamline of the ESRF, Grenoble, France, at a wavelength of λ = 0.206773 Å (60 keV) using a Perkin-Elmer flat panel detector located at 38.5 cm from the sample (Qmax = 24 $Å^{-1}$). The diffraction images were corrected and transformed to 1D diffraction patterns using the PyFAI software[1] up to Qmax = 24 $Å^{-1}$.

For the **1g**, **3a** and **3g** samples, the data were collected using a Bruker kappaCCD diffractometer equipped with an Incoatec IμS microsource for AgKα radiation (λ = 0.5608 Å, 22.1 keV) and a CCD camera located at 10 cm from sample, which was rotated about its axis by 180° during acquisition. 36 images collected every 3° from 2-theta = 0° to 105°. The images were then integrated and averaged to yield a 1D pattern up to Qmax = 17 $Å^{-1}$. The pelletized samples were measured in reflection mode.

Sample **2a** was measured up to Qmax = 17.3 $Å^{-1}$ with a Bruker D8 diffractometer in Debye-Scherrer geometry equipped with MoKα1 radiation selected by a focusing primary Ge(111) monochromator and a 1D LynxEye detector with a 500µm thick Si sensor. The poorer counting statistics lead to a noisier PDF pattern as for the kappaCCD data, as visible in Fig. S5 and S36.

In all cases, data from an empty capillary were also collected for background subtraction and standard samples (LaB_6, CeO_2 and Ni) were used to characterize the instrumental resolution function. The reciprocal space data were converted to PDFs using the PDFgetX3 software.[2] For the laboratory data a damping correction using the Lorch function was applied prior to Fourier transform.

XAS

The sample were prepared by mixing with appropriate amounts of boron nitride (BN) and pressed in to pellet. X-ray absorption spectra were collected at the Aichi Synchrotron Radiation Center (Aichi SR in Japan) on beamline BL5S1. Au foil internal energy calibration was measured simultaneously for each sample. XAS data were background corrected and normalized using the ATHENA software.[3]

XANES Simulation

The XANES spectra of **1c** and **1a** for all the refined models were simulated using finite difference method (FDM) in a fully relativistic frame, including thus the spin-orbit interaction,

as implemented in the FDMNES software. A cluster radius of 7.5 Å around the absorber was used in all calculations, seeing as simulations extended to larger radii gave identical results.

EXAFS refinement.

The Extended X-ray Absorption Fine Structure (EXAFS) regions of the spectra were analyzed via a least-square fitting of the average local structure around gold using the ARTEMIS software.[3] The theoretical paths, scattering amplitude, phase shift and mean free path of the photoelectron were calculated by the FEFF6.0 program embedded in ARTEMIS. Additionally, a E_0 parameter was refined to align the wavenumber grids of the data with those calculated by FEFF.

The number of refined parameters was limited to 9 and 11 variables for the block and chain models respectively, for a fitting range of the Fourier Transform Δk [3:14 $Å^{-1}$] and the range in R over which the fit was evaluated ΔR [1.65:4.4 Å]. K weights of 1, 2 and 3 were simultaneously fitted to distribute the sensitivity of the evaluation of χ^2 over the entire k range and to make better use of the data available. S_0^2 was refined for a metallic gold standard and the refined value of 1 was constrained in the unknown samples.

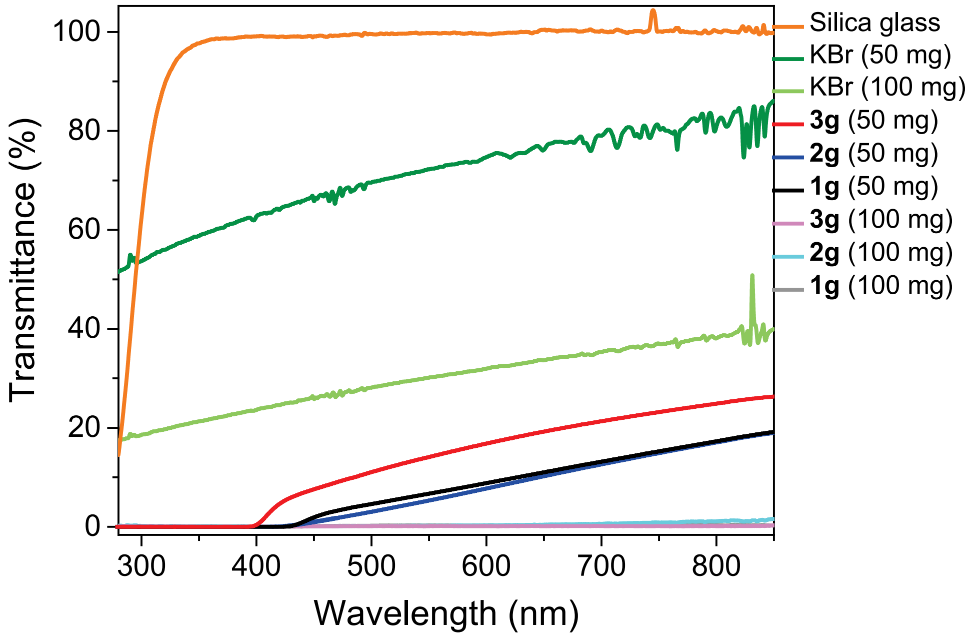

Figure S1. Transmittance curves of the three glass pellets of *g*-$[Au(SR)]_n$ compounds: **1g**, **2g** and **3g** prepared with different masses and compared to a silica glass and KBr pellets.

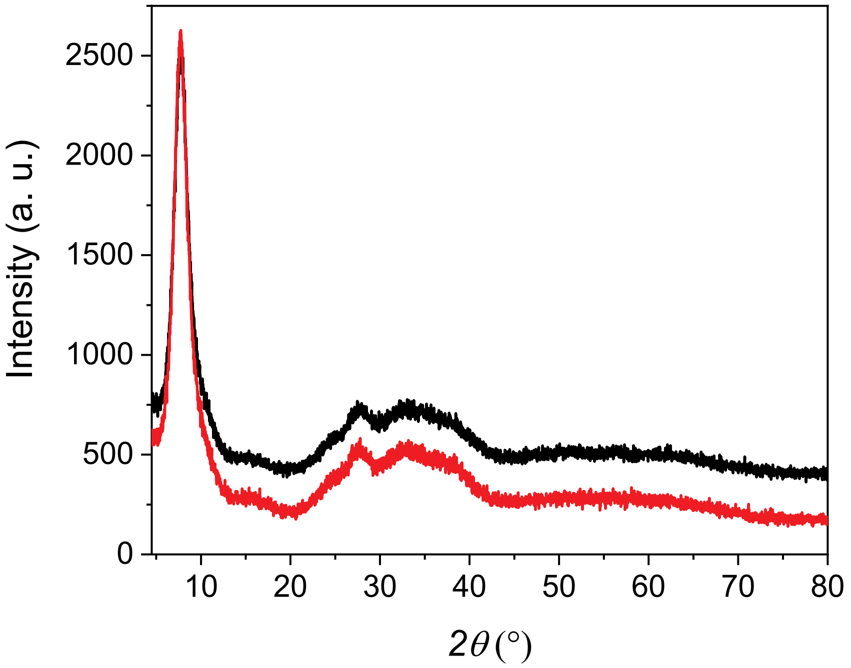

Figure S2. Comparison of the PXRD of the powdery **1a** (black) and glassy **1g** (red) amorphous $[Au(SPh)]_n$.

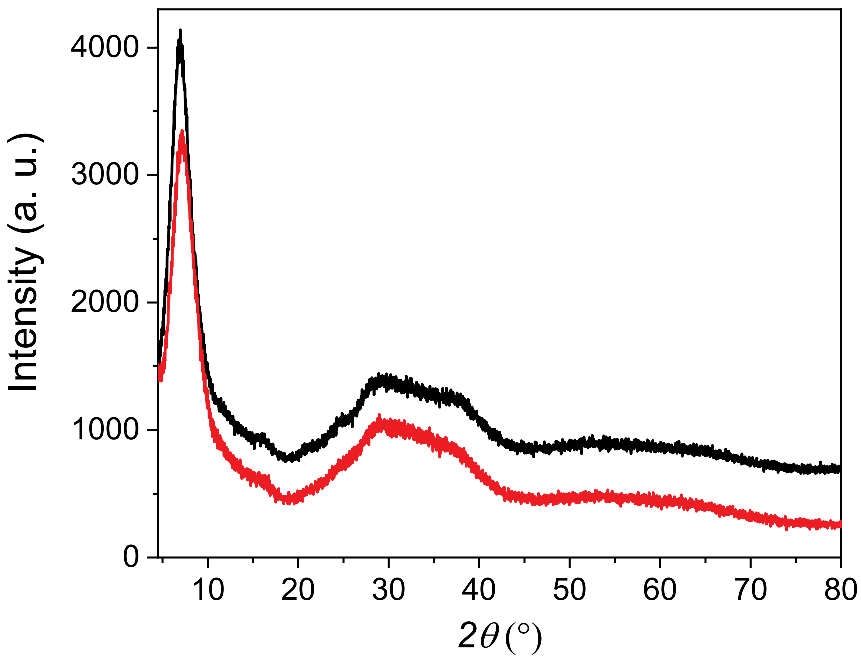

Figure S3. Comparison of the PXRD of the powdery **2a** (black) and glassy **2g** (red) amorphous $[Au(SMePh)]_n$.

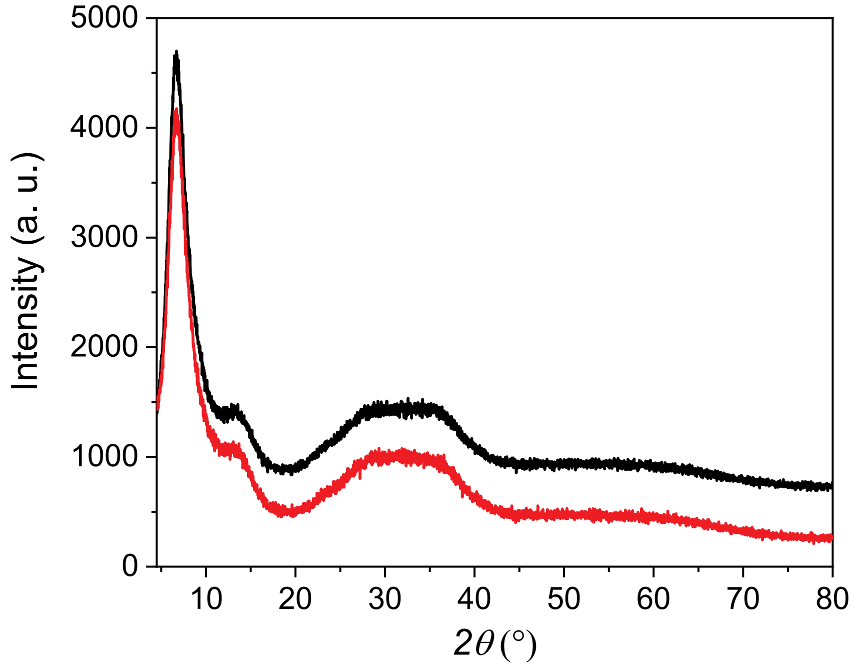

Figure S4. Comparison of the PXRD of the powdery **3a** (black) and glassy **3g** (red) amorphous $[Au(SEtPh)]_n$.

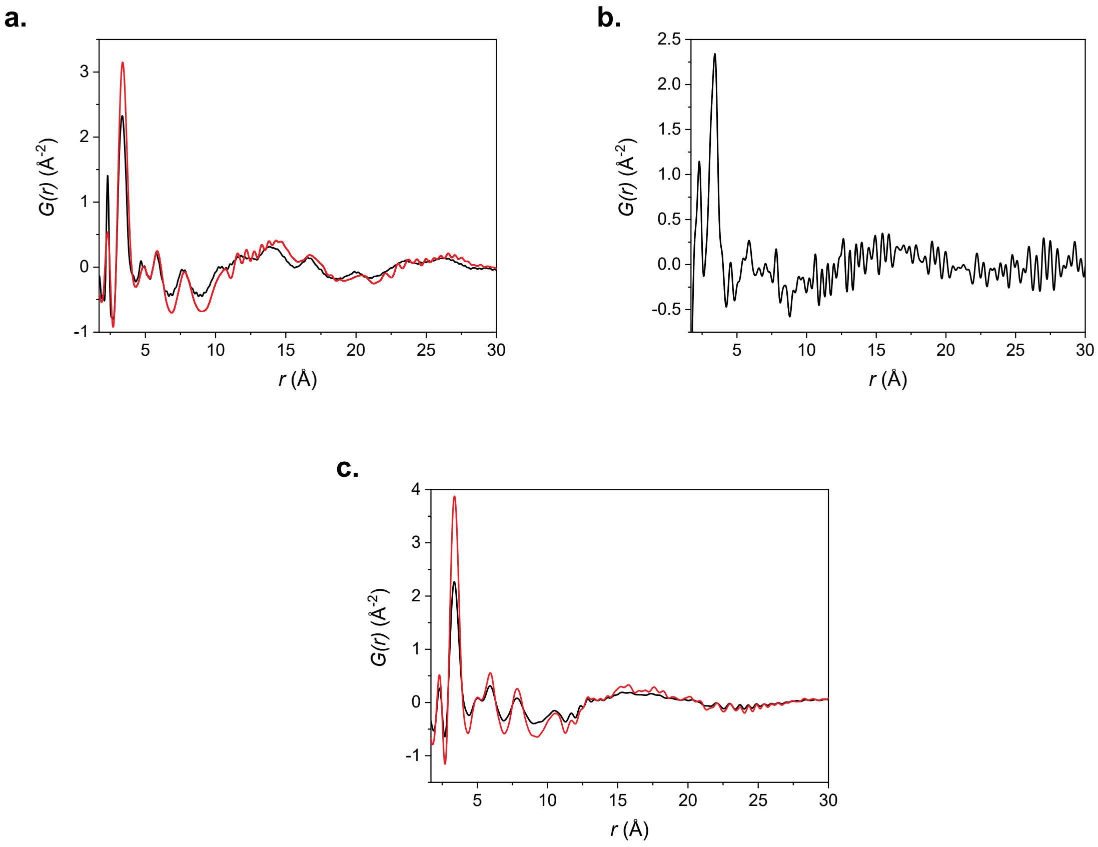

Figure S5. Comparisons of the pair distribution functions *G(r)* of amorphous and pelletized samples: a. $[Au(SPh)]_n$ (**1a** (black, rescaled) and **1g** (red)), b. $[Au(SMePh)]_n$ (**2a** (black)), c. $[Au(SEtPh)]_n$ (**3a** (black) and **3g** (red)). Apparent difference of profiles of **2a** is caused by different instrument resolutions.

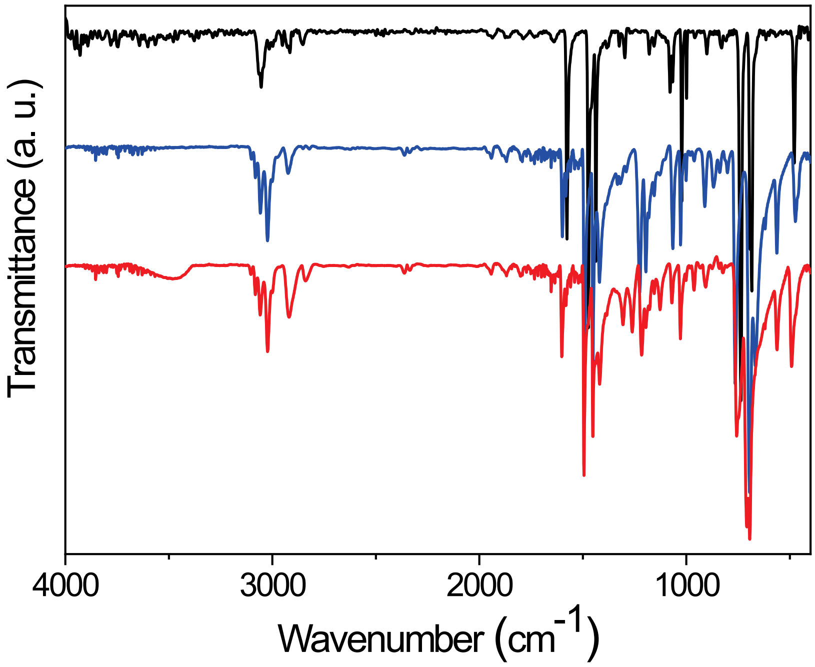

Figure S6. FT-IR spectra of *a*-[Au(SR)]n: **1a** (black), **2a** (blue) and **3a** (red).

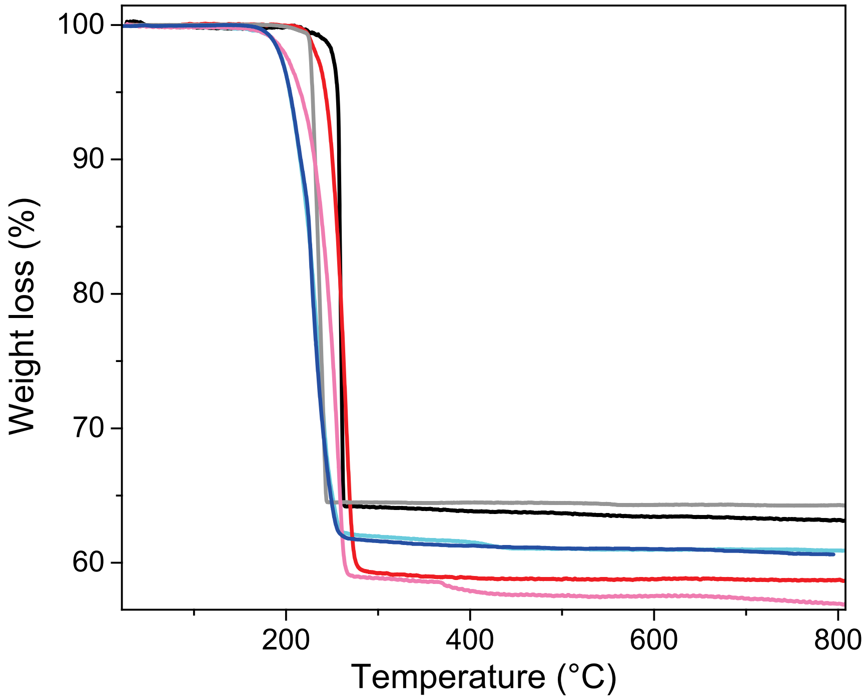

Figure S7. TGA carried out under air at 10°C.min^{-1} of *a*-[Au(SR)]n (**1a** (black), **2a** (blue), **3a** (red)) and *g*-[Au(SR)]n (**1g** (grey), **2g** (sky blue), **3g** (pink)).

Table S1. Temperatures (in °C) of the crystallization (T_C) and the decomposition (T_D) of **1a**, **2a** and **3a** obtained from TGA and DSC experiments carried out at 10°C.min^{-1} under air (in °C).

	T_C	T_D*
**1a**	220	240
**2a**	-	184
**3a**	140	225

* The decomposition temperature is obtained when 0.01 % weight loss is reached.

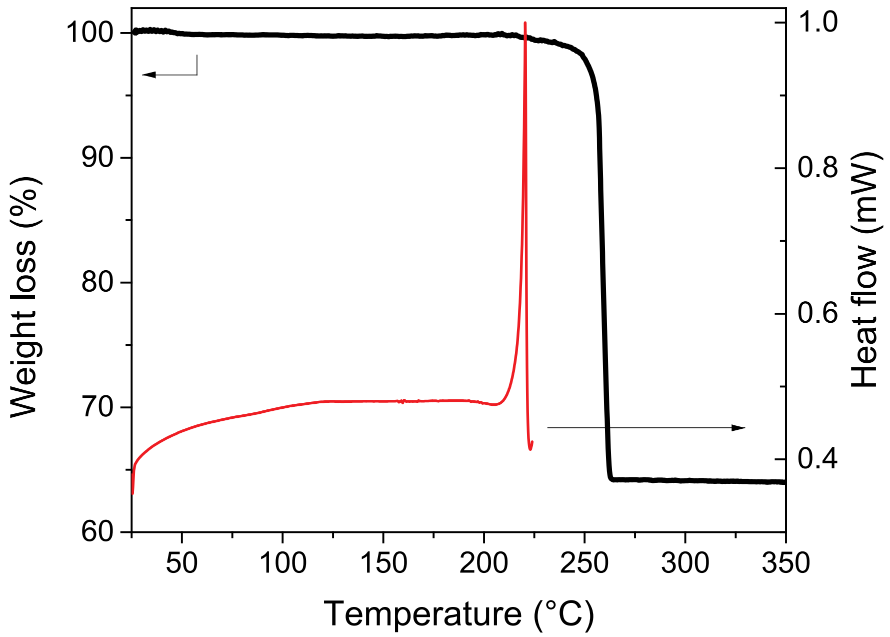

Figure S8. TGA (black) and DSC (red) curves of **1a** carried out at 10 °C.min^{-1}.

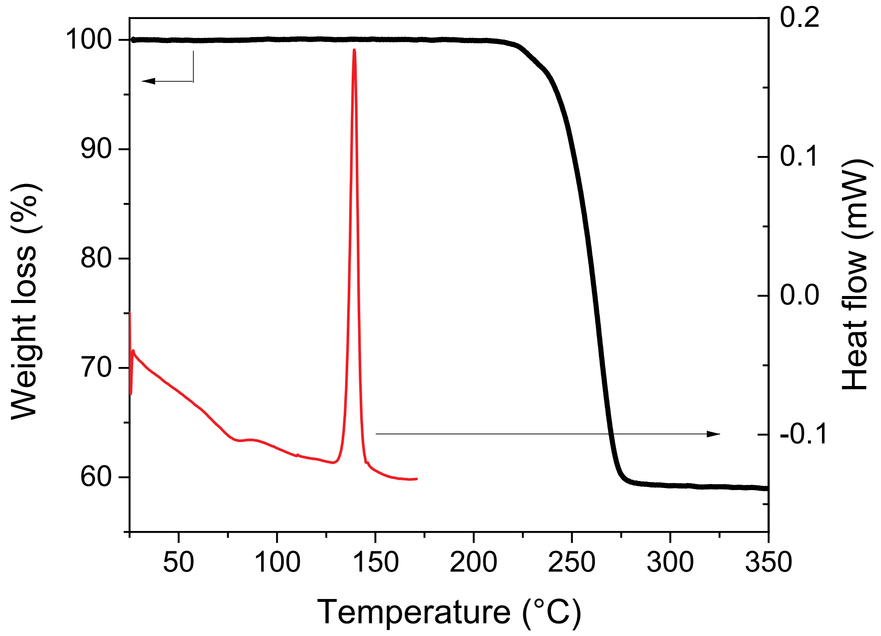

Figure S9. TGA (black) and DSC (red) curves of **3a** carried out at 10 °C.min^{-1}.

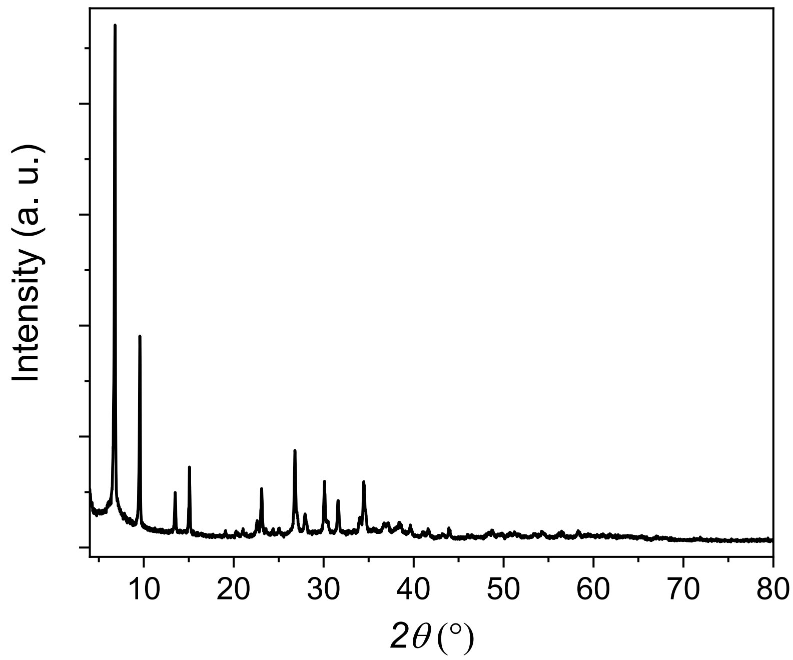

Figure S10. PXRD of the crystalline phase of $[Au(SEtPh)]_n$ obtained by heating **3a**.

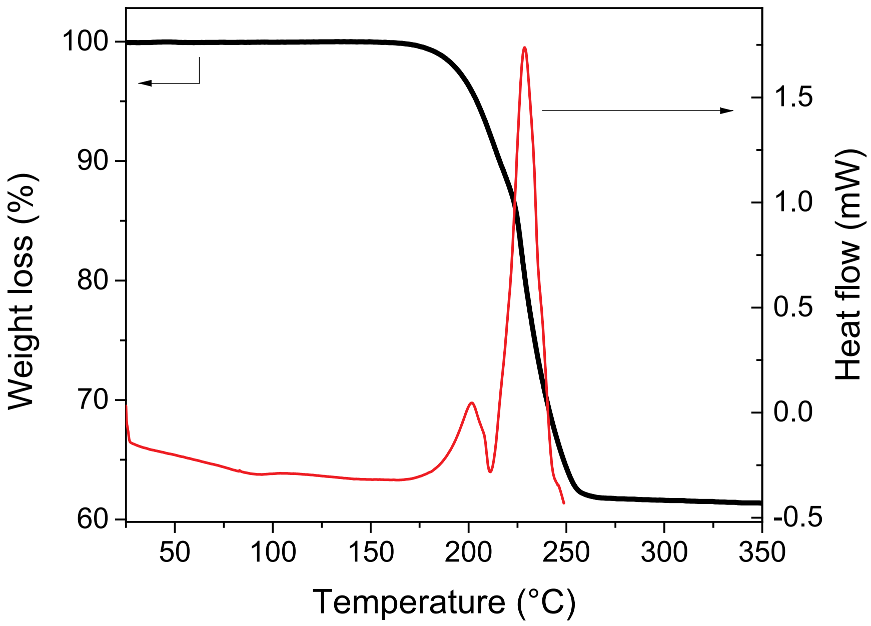

Figure S11. TGA (black) and DSC (red) curves of **2a** carried out at 10 °C.min^{-1}.

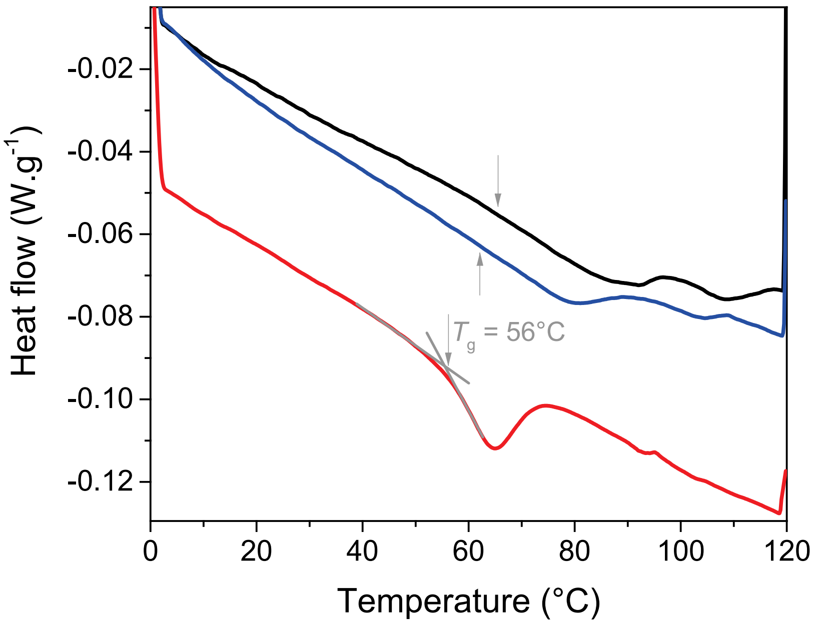

Figure S12. DSC carried out under air at 10 °C.min^{-1} of the three glass pellets of *g*-[Au(SR)]$_n$ compounds: **1g** (black), **2g** (blue) and **3g** (red) with the assignment of its glass transition (T_g).

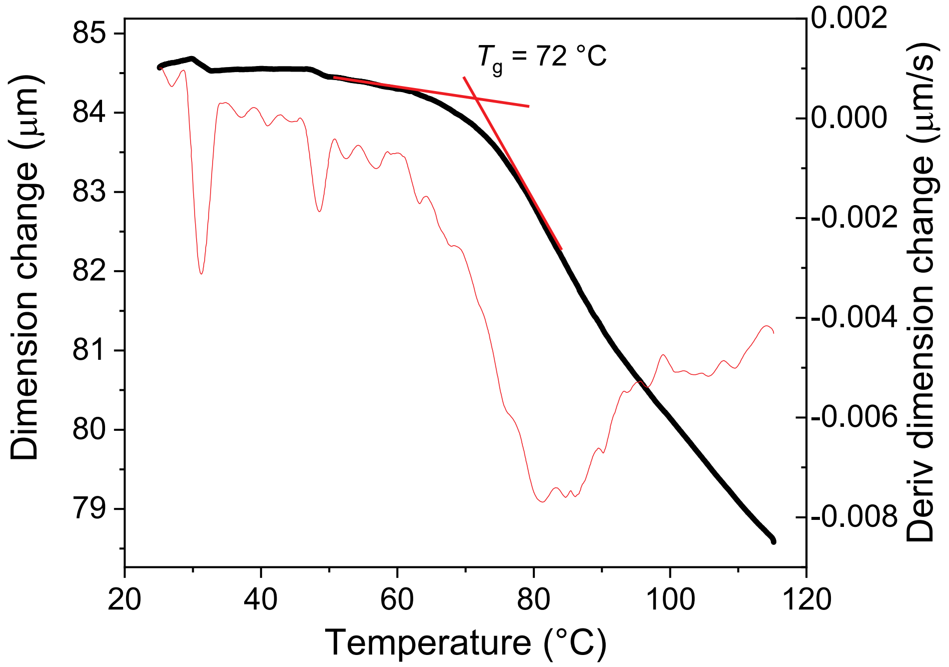

Figure S13. TMA of **1g** (black) and the derivative (red) carried out with a heating rate of 3°C.min^{-1} and a force of 0.1 N under air.

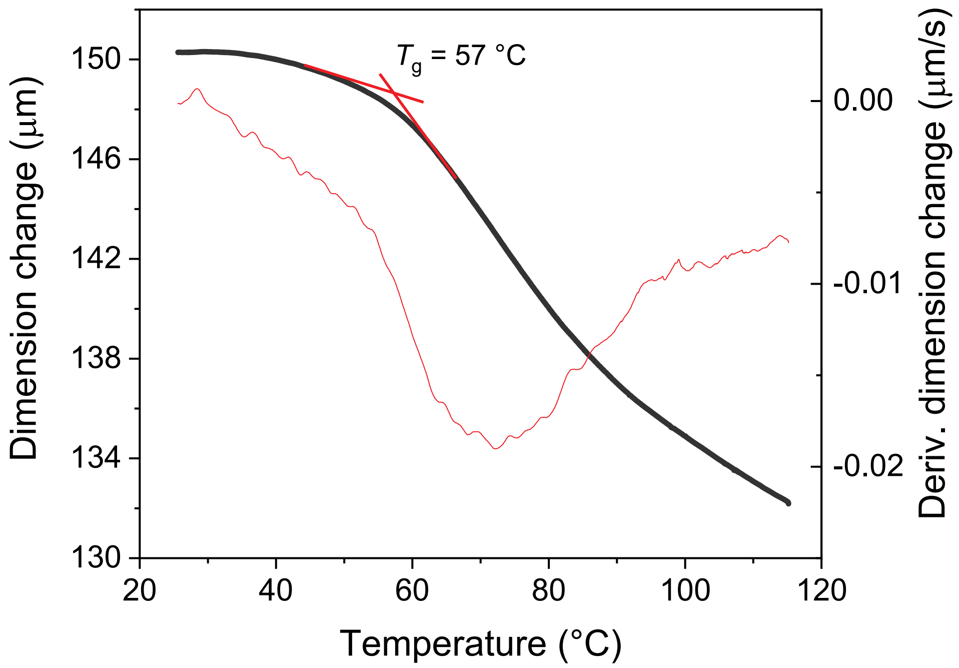

Figure S14. TMA of **2g** (black) and the derivative (red) carried out with a heating rate of 3°C.min^{-1} and a force of 0.1 N under air.

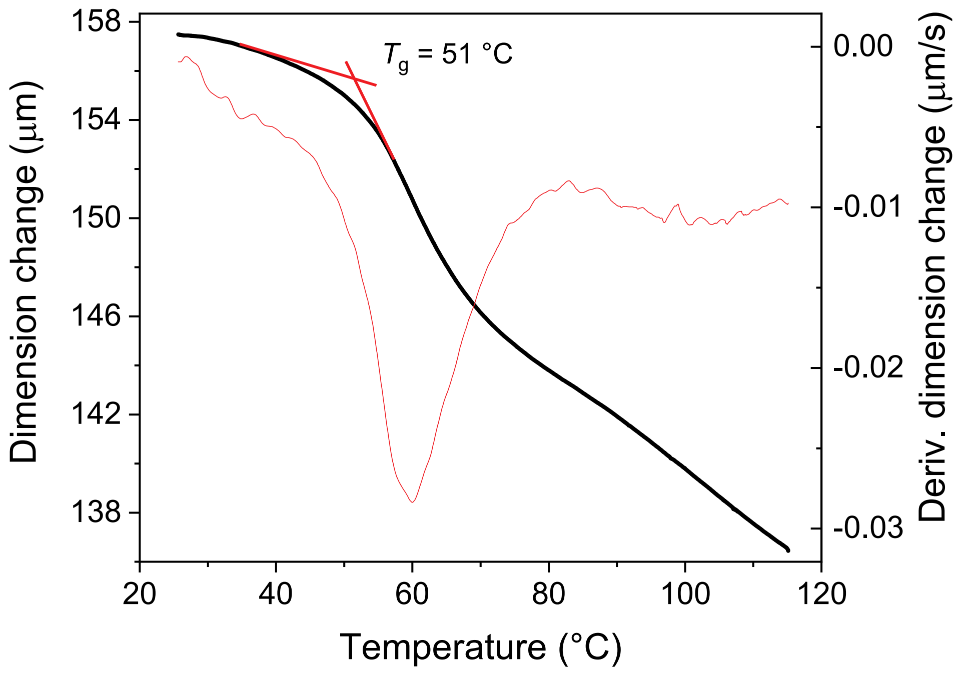

Figure S15. TMA of **3g** (black) and the derivative (red) carried out with a heating rate of 3°C.min^{-1} and a force of 0.1 N under air.

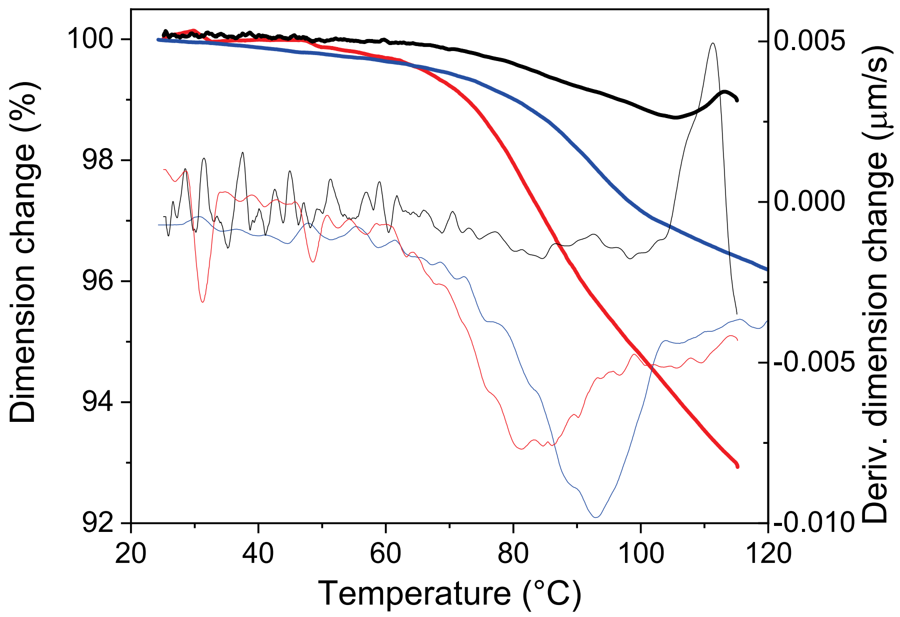

Figure S16. TMA (thick curves) of **1g** and the derivatives (thin curves) carried out under air with different heating rates and applied forces: 3°C.min^{-1} and 0.1 N (red), 5°C.min^{-1} and 0.1 N (blue) and 3°C.min^{-1} and 0.01 N (black).

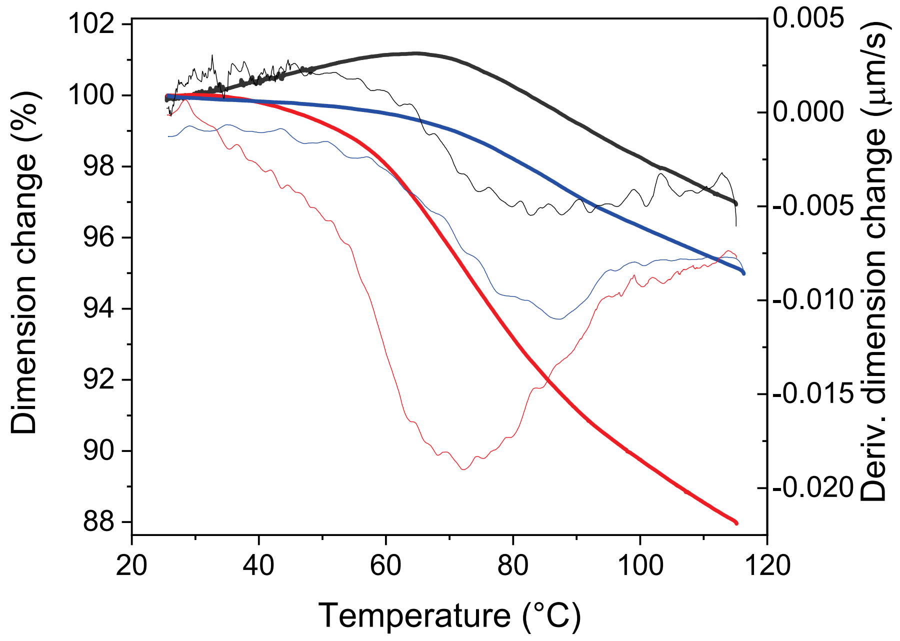

Figure S17. TMA (thick curves) of **2g** and the derivatives (thin curves) carried out under air with different heating rates and applied forces: 3°C.min^{-1} and 0.1 N (red), 5°C.min^{-1} and 0.1 N (blue) and 3°C.min^{-1} and 0.01 N (black).

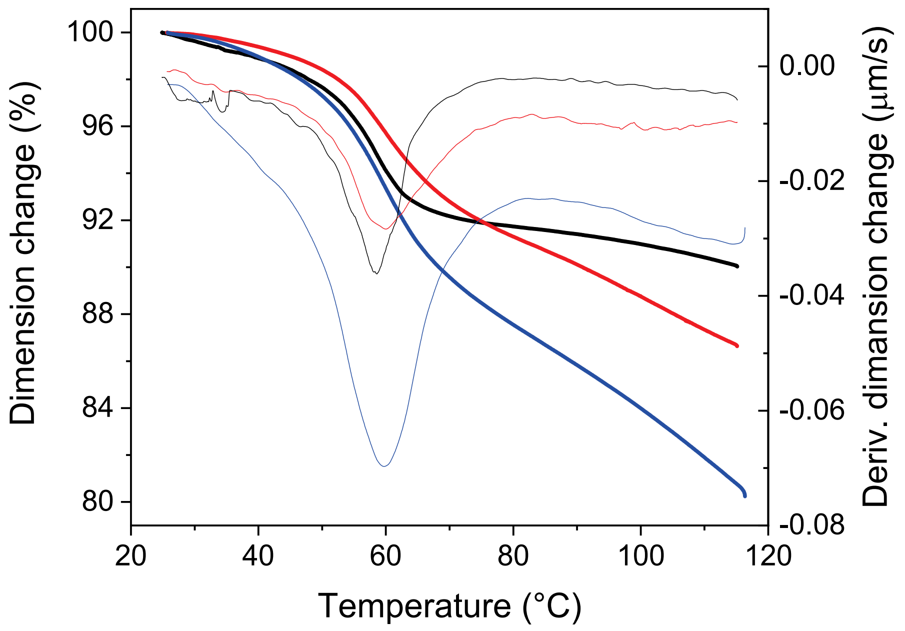

Figure S18. TMA (thick curves) of **3g** and the derivatives (thin curves) carried out under air with different heating rates and applied forces: 3°C.min^{-1} and 0.1 N (red), 5°C.min^{-1} and 0.1 N (blue) and 3°C.min^{-1} and 0.01 N (black).

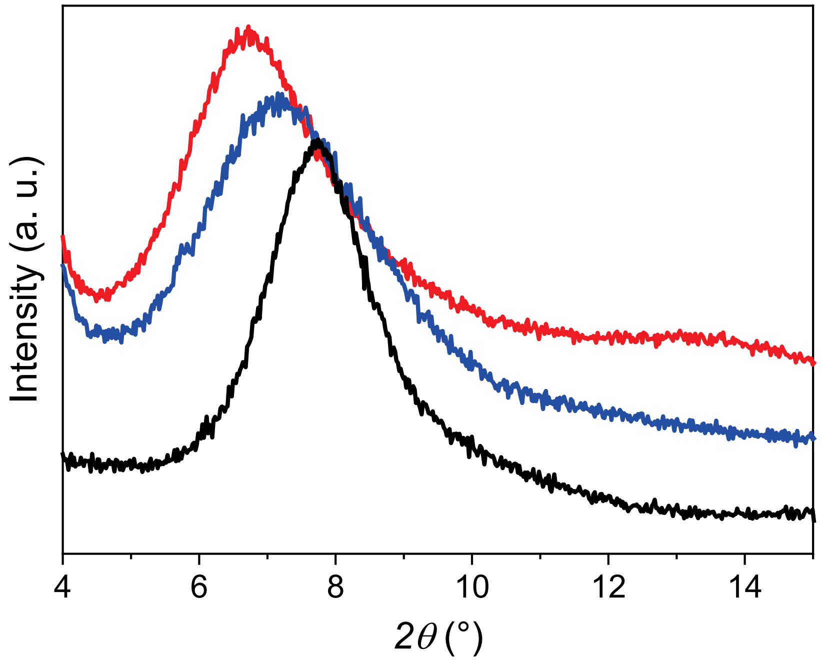

Figure S19. Zoom on the powder XRD patterns of the three amorphous $[Au(SR)]_n$ compounds: **1g** (black), **2g** (blue) and **3g** (red).

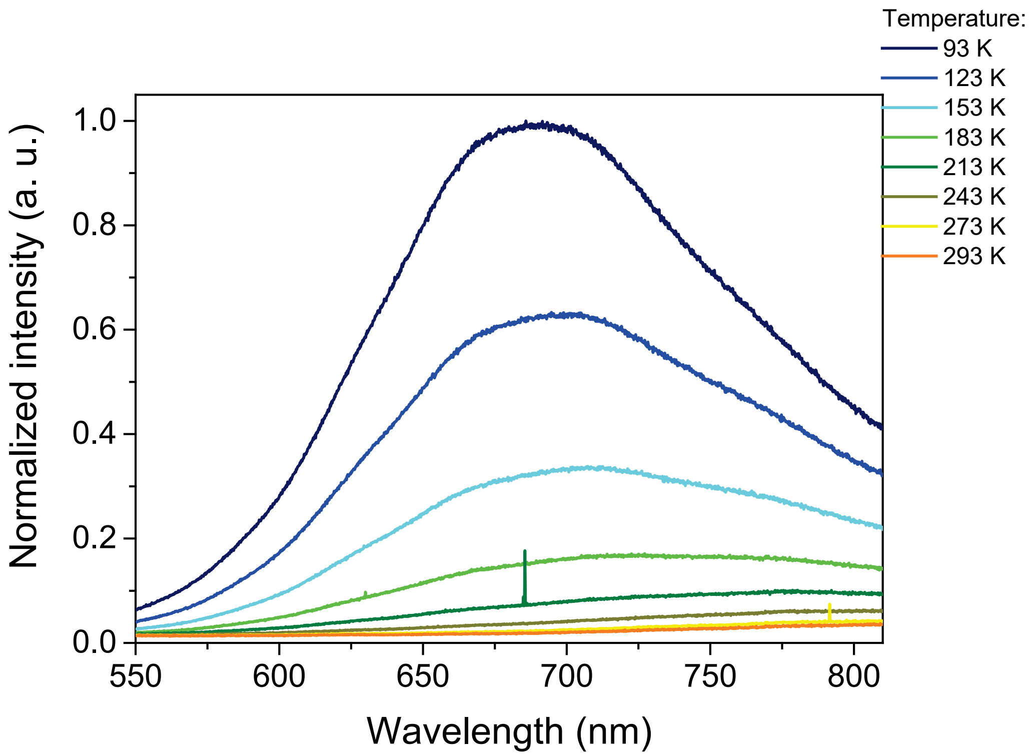

Figure S20. Emission spectra (λ_{ex} = 412 nm) of **1a** in the solid state with the temperature.

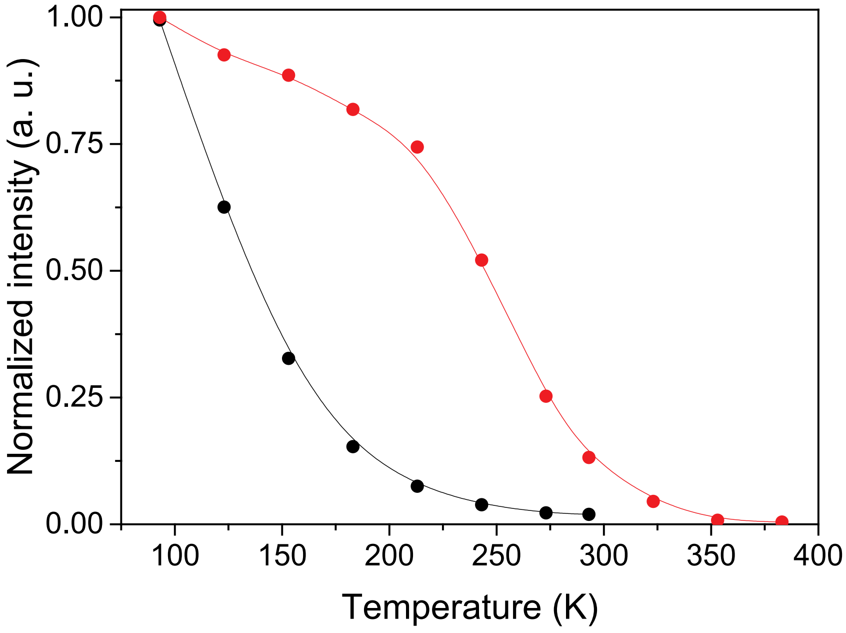

Figure S21. Maximum of emission intensities of **1a** (black, λ_{ex} = 412 nm, λ_{em} = 695 nm) and **1c** (red, λ_{ex} = 320 nm, λ_{em} = 675 nm) with the temperature.

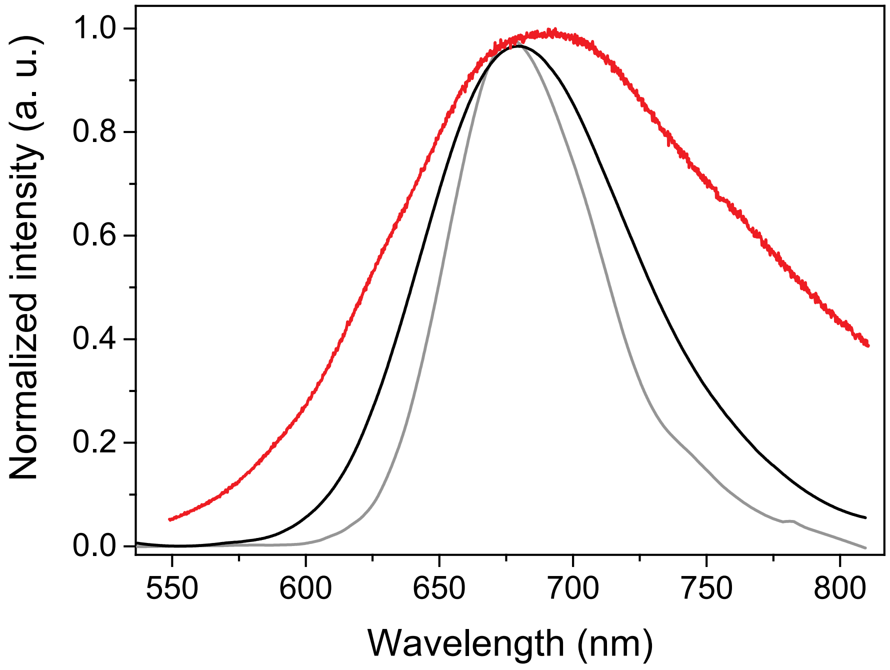

Figure S22. Emission-excitation spectra of **1c** at 293 K (black, λ_{ex} = 320 nm, λ_{em} = 675 nm) and at 93 K (grey, λ_{ex} = 260 nm, λ_{em} = 675 nm) and **1a** at 93 K (red, λ_{ex} = 412 nm, λ_{em} = 690 nm) carried out in solid state.

Table S2. Emission, excitation wavelengths and Stokes shift of the studied gold thiolate compounds.

Product		Excitation, nm	Emission, nm	Stokes shift, cm^{-1}
**1**	**1c**	320	675	16435
	**1a**	412	690	9779
	**1g**	420	690	9317
**2**	**2a**	372	675	12067
	**2g**	380	675	11501
**3**	**3a**	344	655	13803
	**3g**	396	620	9123
		360	655	12511

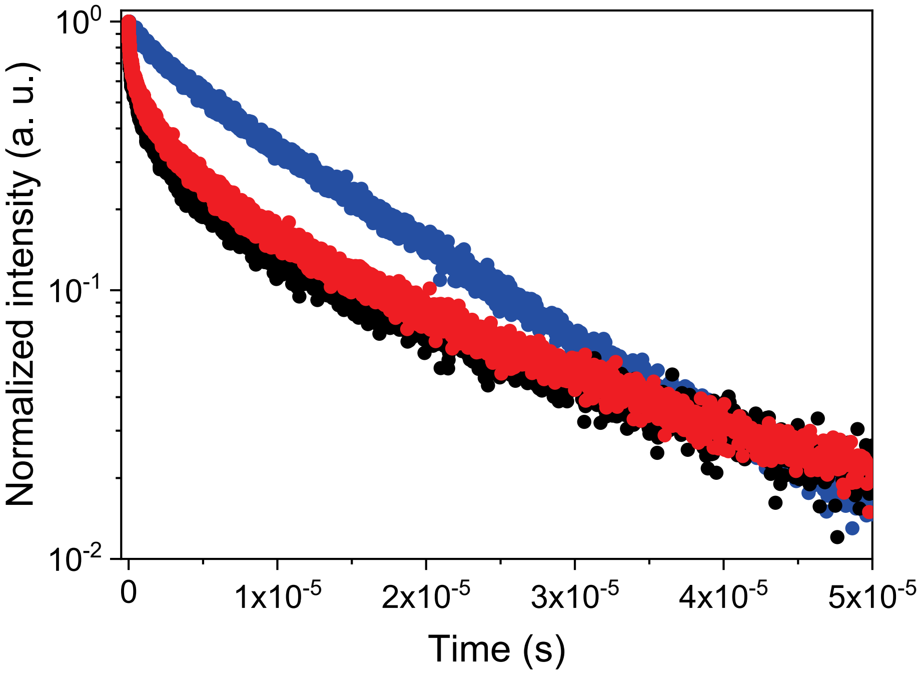

Figure S23. Lifetime decays of **1a** (black), **1g** (red) and **1c** (blue) at 93 K.

Table S3. Luminescence lifetimes (τ_i), their pre-exponential factors (a_i), parameters (β_i), average lifetimes ($<\tau_i>$) and contributions of $[Au(SR)]_n$ at 93 K.

Compound	$[Au(SPh)]_n$			$[Au(SMePh)]_n$		$[Au(SEtPh)]_n$	
	1c	1a	1g	2a	2g	3a	3g
(λ_{em}, λ_{exc}), nm	(332, 675)	(412, 690)	(420, 690)	(372, 675)	(380, 675)	(344, 655)	(340, 655)
Stokes shift, cm^{-1}	15306	9779	9317	12067	11501	13803	14145
$a_1 \pm \sigma a_1$	0.9782 ± 0.0270	0.1961 ± 0.0074	0.2897 ± 0.0246	0.1393 ± 0.0013	0.4942 ± 0.0031	0.3873 ± 0.0050	0.4143 ± 0.0055
$\tau_1 \pm \sigma\tau_1$	8.37E-06 ± 1.27E-07	9.86E-06 ± 3.44E-07	8.52E-06 ± 3.99E-07	8.71E-07 ± 1.89E-08	1.19E-06 ± 1.86E-08	7.72E-06 ± 1.01E-07	6.82E-06 ± 9.50E-08
$\beta_1 \pm \sigma\beta_1$	0.783 ± 0.006	0.642 ± 0.007	0.628 ± 0.007	0.504 ± 0.003	0.543 ± 0.003	0.879 ± 0.005	0.861 ± 0.005
$<\tau_1> \pm <\sigma\tau_1>$	9.63E-06 ± 1.62E-07	1.37E-05 ± 4.97E-07	1.21E-05 ± 5.83E-07	1.72E-06 ± 4.00E-08	2.07E-06 ± 3.46E-08	8.23E-06 ± 1.20E-07	7.36E-06 ± 1.14E-07
Contribution 1, %	98	71	85	88	93	82	84
$a_2 \pm \sigma a_2$	0.0183 ± 0.0296	0.6468 ± 0.0095	0.4362 ± 0.0321	3.58E-04 ± 1.04E-05	1.19E-03 ± 5.43E-05	0.6923 ± 0.0108	0.5291 ± 0.0118
$\tau_2 \pm \sigma\tau_2$	6.42E-06 ± 6.32E-06	1.38E-07 ± 1.65E-08	4.13E-07 ± 5.04E-08	7.30E-05 ± 1.86E-06	4.02E-05 ± 1.91E-06	2.24E-07 ± 6.27E-09	2.38E-07 ± 7.06E-09
$\beta_2 \pm \sigma\beta_2$	0.564 ± 0.096	0.309 ± 0.004	0.398 ± 0.016	0.732 ± 0.005	0.594 ± 0.007	0.360 ± 0.001	0.359 ± 0.001
$<\tau_2> \pm <\sigma\tau_2>$	1.05E-05 ± 1.05E-05	1.12E-06 ± 1.44E-07	1.39E-06 ± 2.04E-07	8.87E-05 ± 2.36E-06	6.13E-05 ± 3.00E-06	1.03E-06 ± 3.04E-08	1.09E-06 ± 3.42E-08
Contribution 2, %	2	29	15	12	7	18	16
R^2	0.99895	0.99756	0.99913	0.99659	0.99664	0.99813	0.99781

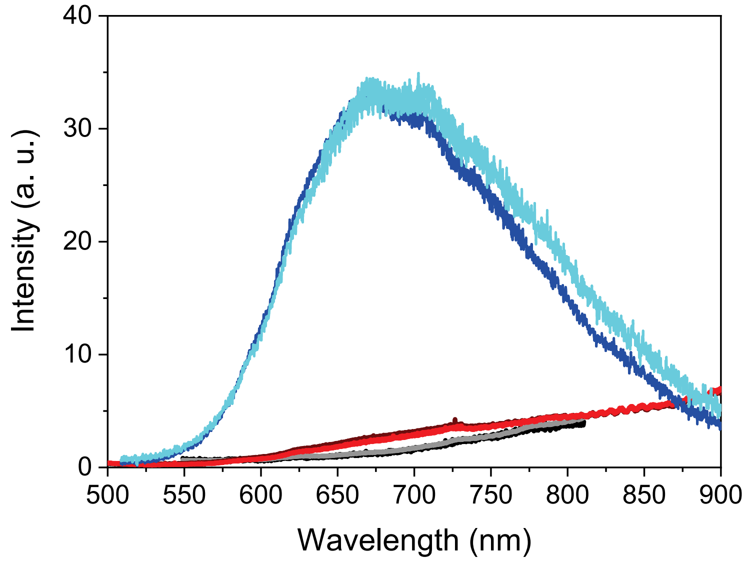

Figure S24. Emission at 293 K of the amorphous gold thiolates as powder and glass forms, where λ_{ex} = 412 nm for **1a** (black), λ_{ex} = 420 nm for **1g** (grey), λ_{ex} = 372 nm for **2a** (blue), λ_{ex} = 380 nm for **2g** (sky blue), λ_{ex} = 340 nm for **3a** (dark red) and **3g** (red).

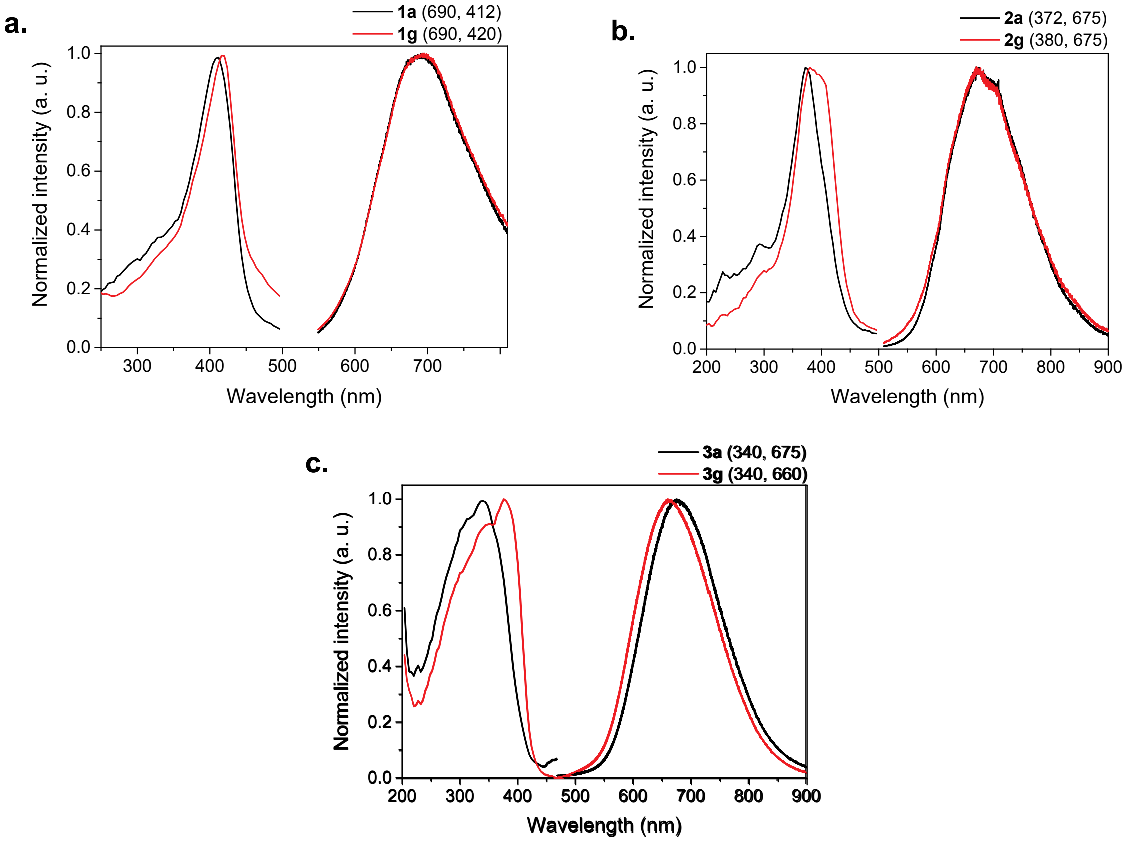

Figure S25. Comparisons of the emission-excitation spectra of the amorphous and pelletized samples carried out in the solid state at 93 K with (λ_{ex}, λ_{em},) in nm: a. $[Au(SPh)]_n$ (**1a** and **1g**), b. $[Au(SMePh)]_n$ (**2a** and **2g**), c. $[Au(SEtPh)]_n$ (**3a** and **3g**).

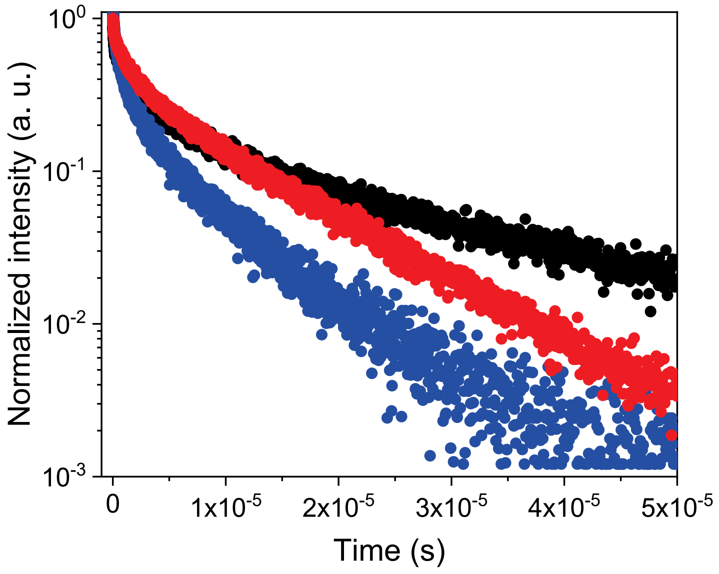

Figure S26. Lifetime decays of **1a** (black), **2a** (blue) and **3a** (red) at 93 K.

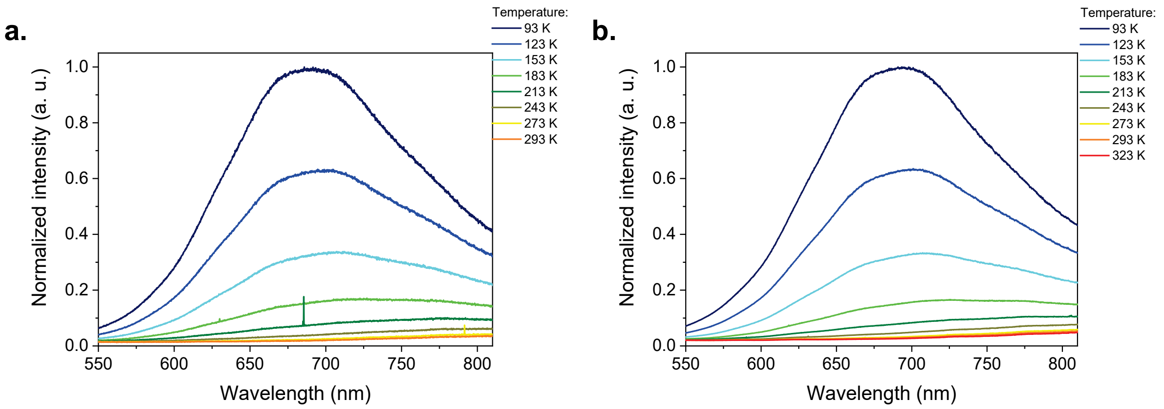

Figure S27. Solid state emission spectra with the temperature of a. **1a** (λ_{exc} = 412 nm) and b. **1g** (λ_{exc} = 420 nm).

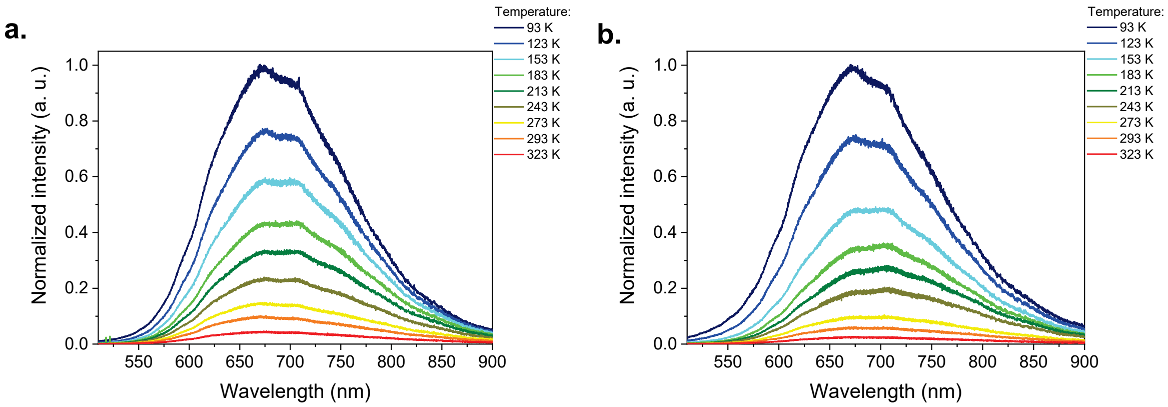

Figure S28. Solid state emission spectra with the temperature of a. **2a** (λ_{exc} = 372 nm) and b. **2g** (λ_{exc} = 380 nm).

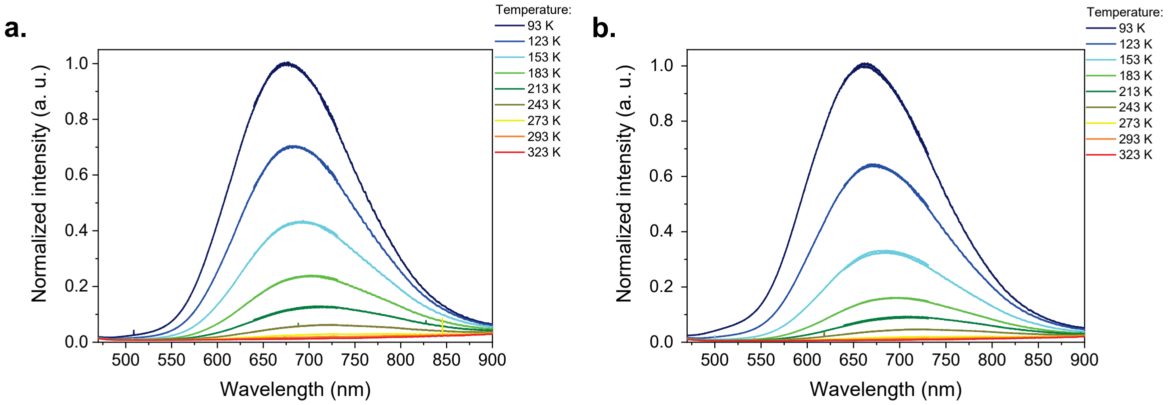

Figure S29. Solid state emission spectra with the temperature of a. **3a** (λ_{exc} = 340 nm) and b. **3g** (λ_{exc} = 340 nm).

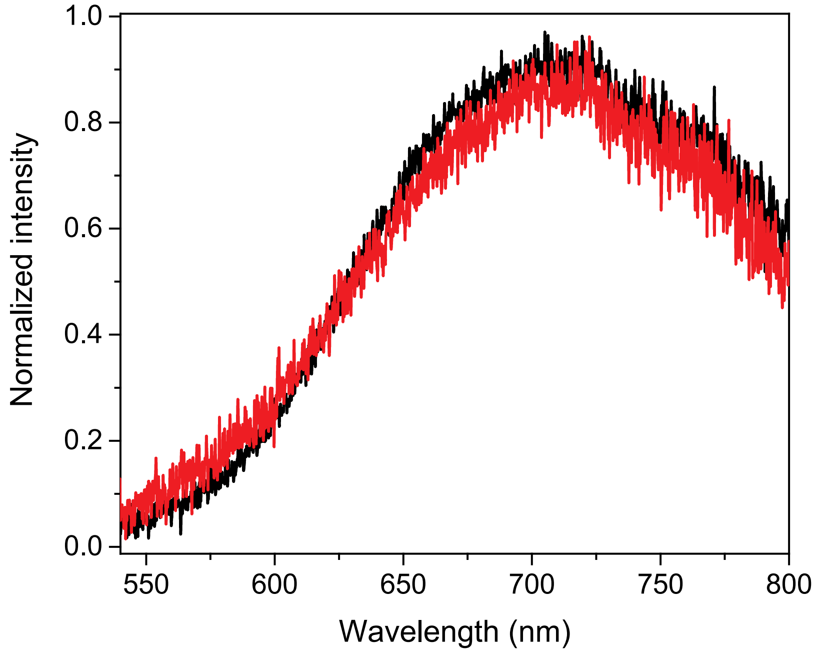

Figure S30. Solid state emission spectra (λ_{exc} = 380 nm) of **3g** carried out at RT on a pellet of 1.5 years old (black) and 2 days old (red).

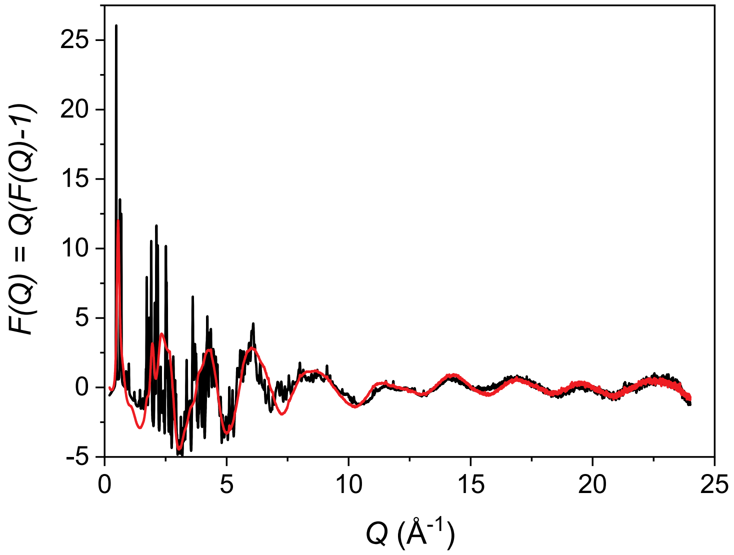

Figure S31. Reduced structure function *F(Q)* for samples **1a** (black) and **1c** (red).

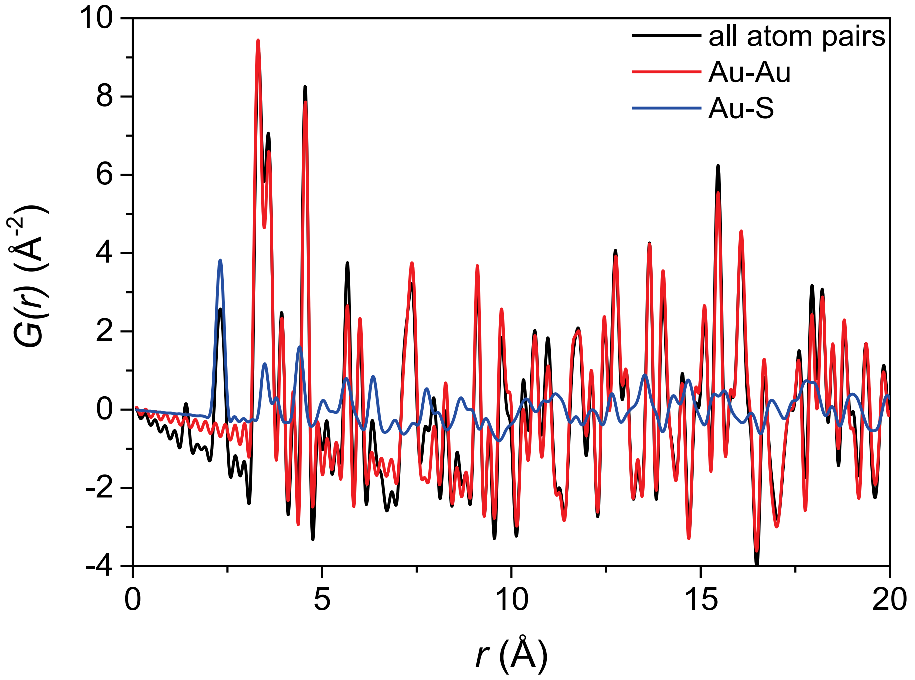

Figure S32. Comparison of simulated PDF for sample **1c** using all atom pairs (black), Au-Au pairs only (red) and Au-S pairs only (blue).

a)

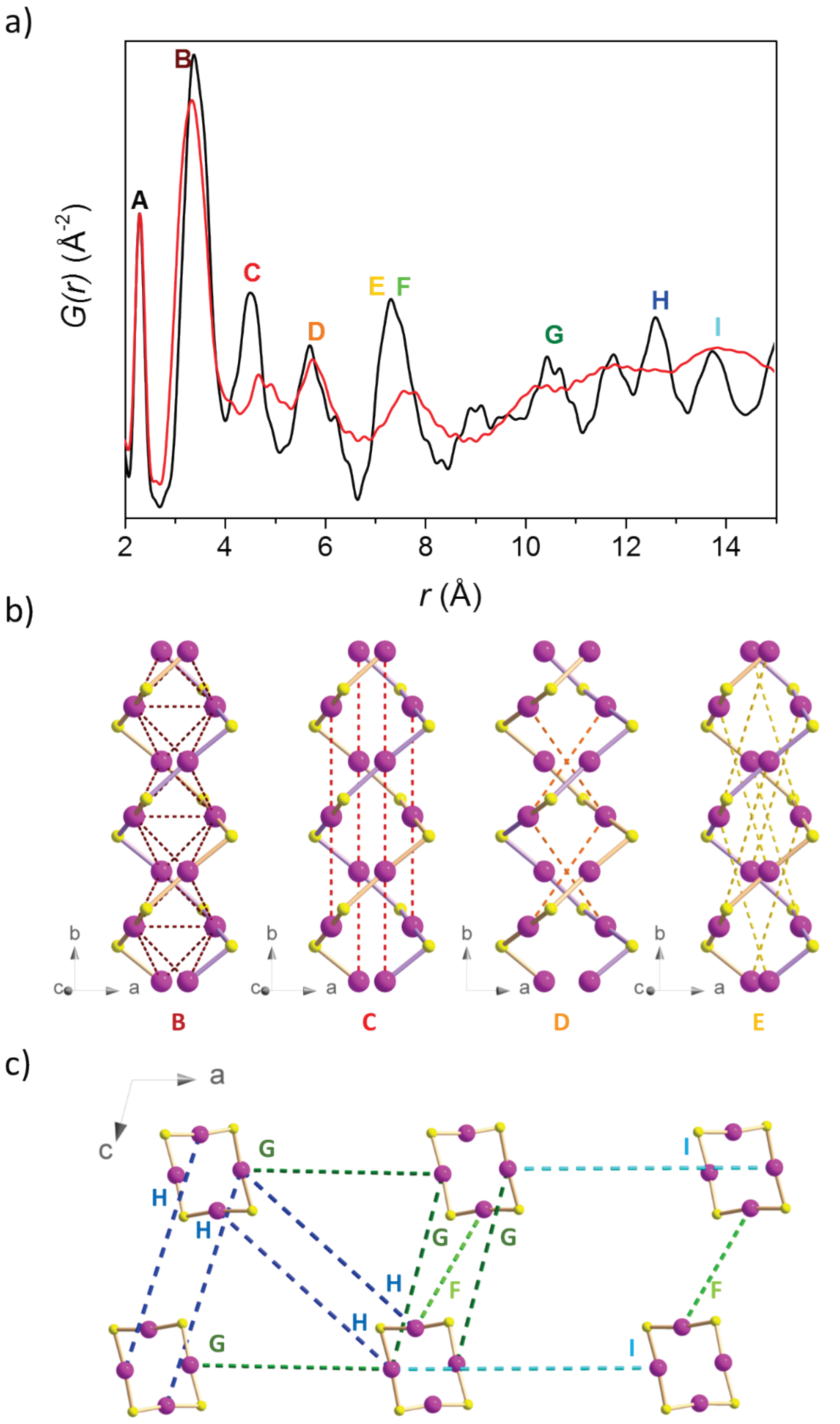

Figure S33. a. PDF data for **1a** and **1c** samples. B-E labels of peaks correspond to the indicated Au-Au distances in b. the double helix, while F-I correspond to some distances between c. Au atoms of neighboring helices. Pink, yellow and grey spheres are for gold, sulfur and carbon atoms. Hydrogen atoms are omitted for clarity.

Table S4. Main Au-S and Au-Au distances (Å) at short *r*-range from PDF data, MolPDF and EXAFS refinements.

Distance	Atoms' pair	1c				1a		
		Reported average structure[4]	Observed PDF	Model refined in MolPDF	EXAFS	Observed PDF	Model refined in MolPDF	EXAFS
**A**	Au1-S1<br>Au1-S2<br>Au2-S1<br>Au2-S2	2.332(2)<br>2.289(2)<br>2.313(2)<br>2.345(2)	2.31(1)	2.32(1)<br>2.28(1)<br>2.31(2)<br>2.33(2)	2.282(9)	2.32(1)	2.32(5)<br>2.27(4)<br>2.36(3)<br>2.28(5)	2.284(3)
**B**	Au1-Au2<br>Au1-Au2$^{\times}$<br>Au1-Au1$^{\times}$<br>Au1-Au2$^{\times}$<br>Au1-Au2	3.323(3)<br>3.331(3)<br>3.369(3)<br>3.603(3)<br>3.610(3)	3.32(15)<br>3.53(18)<br>3.64(8)	3.316(8)<br>3.555(8)<br>3.359(9)<br>3.456(8)<br>3.685(9)	3.34(5)<br>3.51(9)	3.20(4)<br>3.52(4)	3.19(3)<br>3.55(3)<br>3.27(4)<br>3.34(3)<br>3.68(3)	3.3(2)<br>3.5(4)
**C**	Au2-Au2$^{\times}$<br>Au1-Au1$^{\times}$<br>Au2-Au2$^{\times}$	4.031(3)<br>4.533(4)	4.16(6)<br>4.54(4)	4.156(6)<br>4.524(8)	4.45(4)	4.77(4)$^{\lozenge}$	3.81(3)<br>4.69(3)	4.45(4)
**D**	Au1-Au1<br>Au2-Au2	5.648(4)<br>6.066(3)	5.71(9)	5.635(8)<br>6.143(7)		5.81(1.60)$^{\lozenge}$	5.71(3)<br>6.04(3)	

$^{\times}$ Interchain Au-Au distances; $^{\lozenge}$ obtained from the weighted arithmetic mean of several peaks.

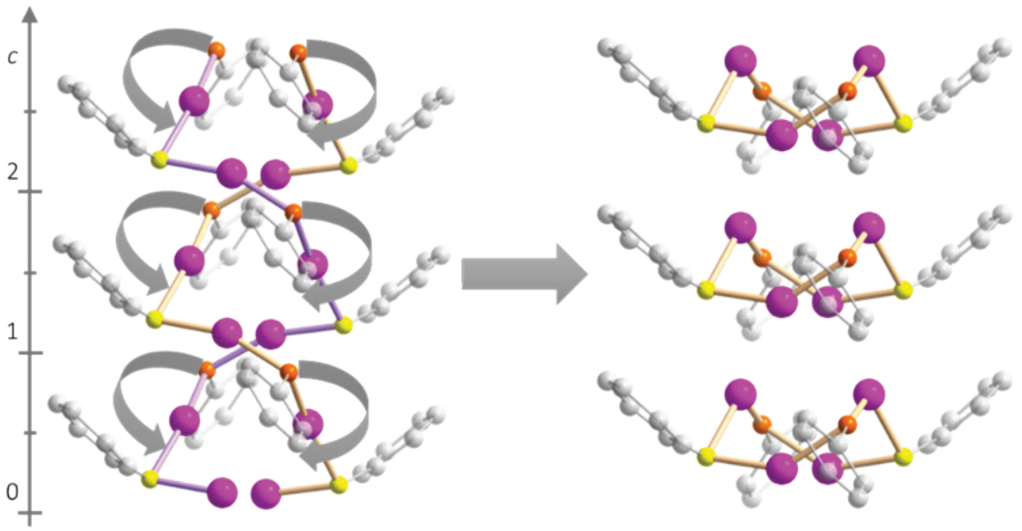

Figure S34. Schematic representation of the tetramer ring model transformation from the double-helix model of $[Au(SPh)]_n$. Pink, yellow and grey spheres are for gold, sulfur and carbon atoms.

Table S5. Bonds and angles observed for the double-helix and tetramer ring models used for the PDF refinements.

	**Double-helix model**	**Tetramer ring model**
**Au-S, Å**	2.28(4)<br>2.28(5)<br>2.31(5)<br>2.36(3)	1.92(6)<br>2.31(5)<br>2.334(4)<br>2.36(3)
**Au-S-Au, °**	86(2)<br>108(2)	86(2)<br>103(2)
**S-Au-S, °**	159(2)<br>166(2)	104(2)<br>140(2)

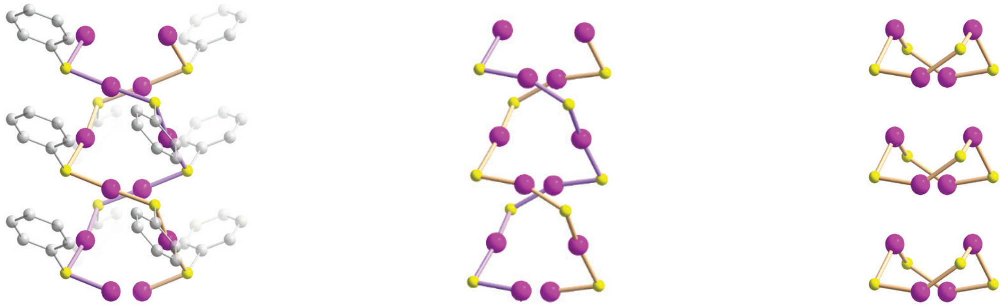

a. **b.** **c.**

Figure S35. Comparisons of the crystalline structure of a. **1c** and the models obtained by PDF refinements with MolPDF software for **1a** sample by using b. the double-helix and c. the tetramer ring models. Pink, yellow and grey spheres are for gold, sulfur and carbon atoms.

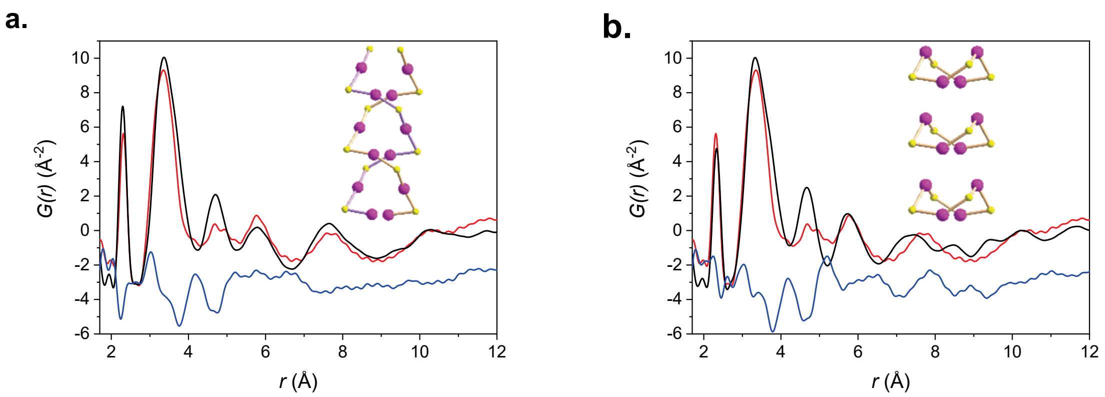

Figure S36. Results of the PDF refinement using the MolPDF software for the **1a** sample using a. double-helix and b. tetramer ring models (shown in the insets; pink and yellow spheres are for gold and sulfur, organic ligands are omitted): experiment (red), calculation (black) and difference (blue).

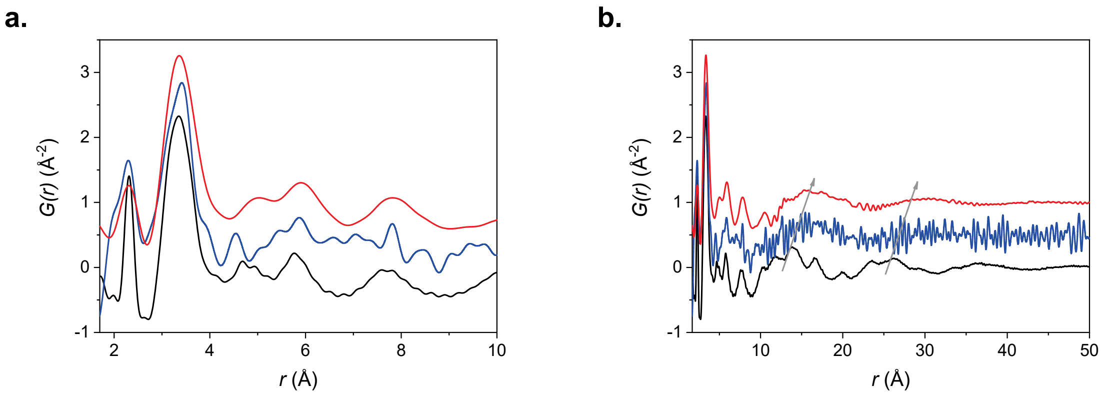

Figure S37. Comparison of the pair distribution functions *G(r)* of samples a. **1a*** (black), **2a** (blue) and **3a** (red); b. the long *r* part of the PDFs up to 50 Å, arrows are indicating shift of the position of broad features corresponding to the correlations between the double helices. Apparent difference of profiles is caused by different instrument resolutions. * rescaled

Table S6. Best-fit interatomic bond distances ‘R’, mean-square atomic displacements ‘σ^2’ and path degeneracy ‘N’ for samples **1c** and **1a**.

	**1c**			**1a** Double-helix model		
Rw (%)	3.0			2.7		
	R (Å)	σ^2 (Å^2)	N	R (Å)	σ^2 (Å^2)	N
Au–S1	2.282(9)	0.0030(9)	2.0(2)	2.284(3)	0.0025(7)	1.5(1)
Au–Au1	3.34(5)	0.014(9)	-	3.3(2)	0.03(1)	-
Au–Au2	3.51(9)	0.014(9)	-	3.5(4)	0.03(1)	-
Au–S2	3.98(5)	0.004(3)	-	3.96(6)	0.008(4)	-
Au–S3	4.45(4)	0.004(3)	-	4.45(4)	0.008(4)	-

The paths are denoted as Au–Xn, where X is Au or S and n indicates atomic shells 1-3.

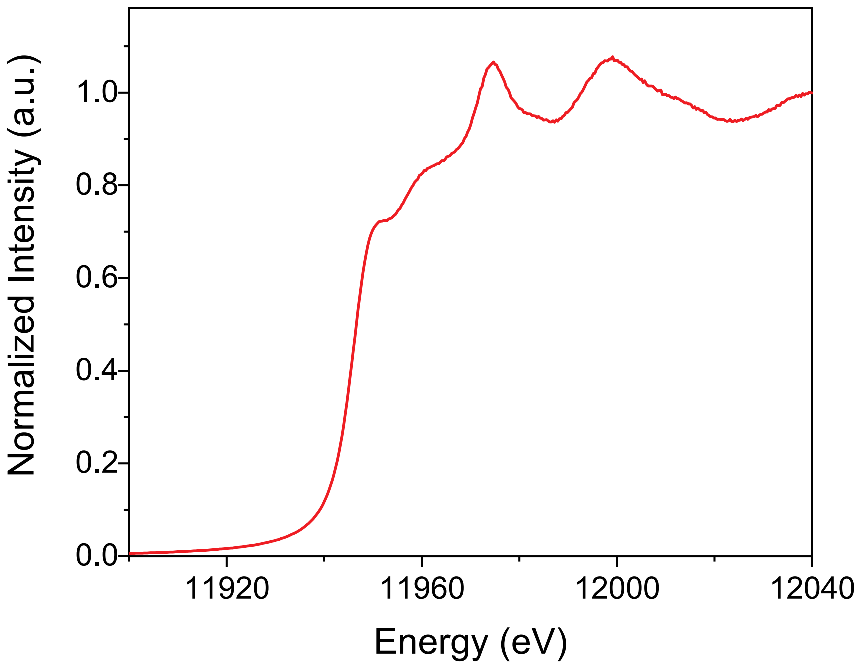

Figure S38. Au L_3-edge XANES of metallic Au foil used as a standard for energy calibrations.

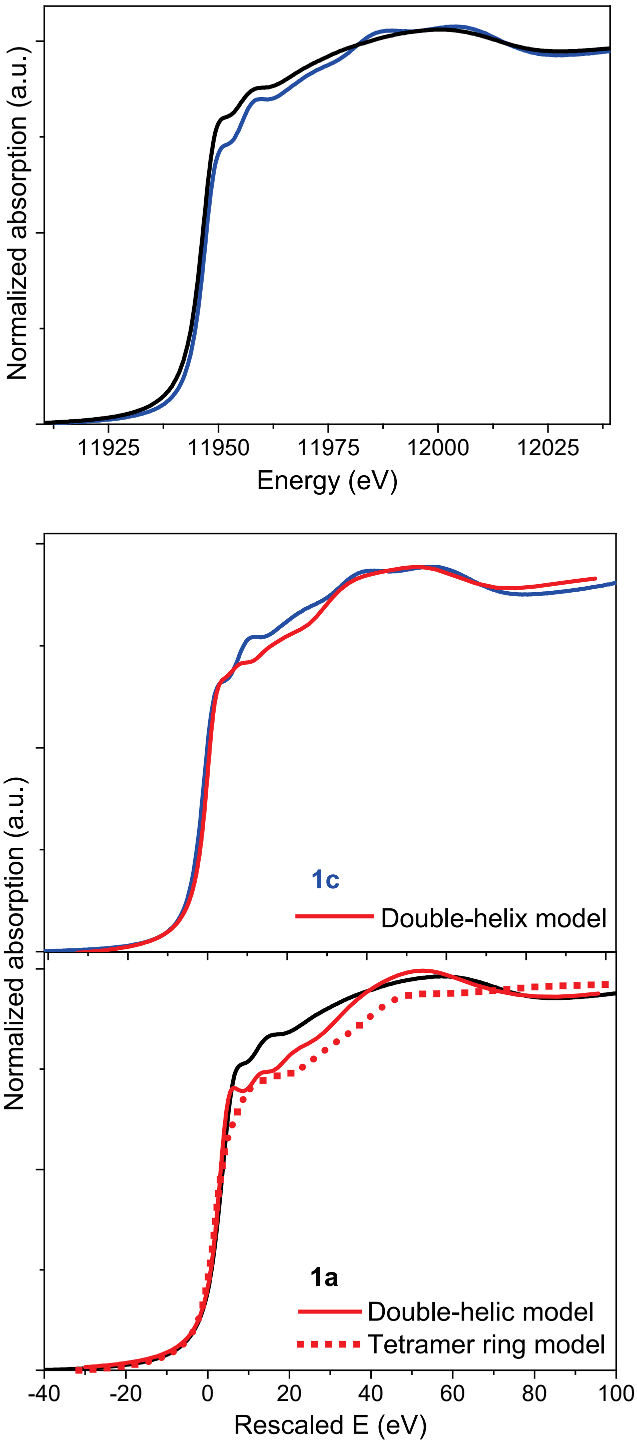

Figure S39. 1c (blue) and **1a** (black) XANES data (top). Comparison between the simulated and experimental XANES for **1c** (middle) and **1a** (bottom). Simulations using the double-helix and tetramer ring models are denoted by solid red and dotted red lines, respectively. For the middle and bottom figures, the rescaled energy values corresponding to the outputs of the FDMNES simulations are plotted in the X-axis.

References:

1. Kieffer, J.; Karkoulis, D., PyFAI, a versatile library for azimuthal regrouping. *J. Phys.: Conf. Ser.* **2013,** *425*, 202012.
2. Juhas, P.; Davis, T.; Farrow, C. L.; Billinge, S. J. L., *PDFgetX3*: a rapid and highly automatable program for processing powder diffraction data into total scattering pair distribution functions. *J. Appl. Cryst.* **2013,** *46*, 560.
3. Ravel, B.; Newville, M., *ATHENA, ARTEMIS, HEPHAESTUS*: data analysis for X-ray absorption spectroscopy using IFEFFIT. *J Synchr. Rad.* **2005,** *12*, 537.
4. Lavenn, C.; Okhrimenko, L.; Guillou, N.; Monge, M.; Ledoux, G.; Dujardin, C.; Chiriac, R.; Fateeva, A.; Demessence, A., A luminescent double helical gold(I)–thiophenolate coordination polymer obtained by hydrothermal synthesis or by thermal solid-state amorphous-to-crystalline isomerization. *J. Mater Chem. C* **2015,** *3*, 4115.
